# Dual-role epitope on SARS-CoV-2 spike enhances and neutralizes viral entry across different variants

**DOI:** 10.1371/journal.ppat.1012493

**Published:** 2024-09-05

**Authors:** Gang Ye, Fan Bu, Ruangang Pan, Alise Mendoza, Divyasha Saxena, Jian Zheng, Stanley Perlman, Bin Liu, Fang Li

**Affiliations:** 1 Department of Pharmacology, University of Minnesota Medical School, Minneapolis, Minnesota, United States of America; 2 Center for Emerging Viruses, University of Minnesota, Minneapolis, Minnesota, United States of America; 3 Department of Microbiology and Immunology, University of Iowa, Iowa City, Iowa, United States of America; 4 Center for Predictive Medicine, University of Louisville, Louisville, Kentucky, United States of America; 5 Department of Microbiology and Immunology, University of Louisville, Louisville, Kentucky, United States of America; 6 Hormel Institute, University of Minnesota, Austin, Minnesota, United States of America; Institut Pasteur, FRANCE

## Abstract

Grasping the roles of epitopes in viral glycoproteins is essential for unraveling the structure and function of these proteins. Up to now, all identified epitopes have been found to either neutralize, have no effect on, or enhance viral entry into cells. Here, we used nanobodies (single-domain antibodies) as probes to investigate a unique epitope on the SARS-CoV-2 spike protein, located outside the protein’s receptor-binding domain. Nanobody binding to this epitope enhances the cell entry of prototypic SARS-CoV-2, while neutralizing the cell entry of SARS-CoV-2 Omicron variant. Moreover, nanobody binding to this epitope promotes both receptor binding activity and post-attachment activity of prototypic spike, explaining the enhanced viral entry. The opposite occurs with Omicron spike, explaining the neutralized viral entry. This study reveals a unique epitope that can both enhance and neutralize viral entry across distinct viral variants, suggesting that epitopes may vary their roles depending on the viral context. Consequently, antibody therapies should be assessed across different viral variants to confirm their efficacy and safety.

## Author summary

Antibodies bind to specific epitopes on viral surface glycoproteins to perform their function: inhibiting viral infection, having no effect, or, in rare cases, enhancing viral infection. In our research, we used nanobodies (small, single-domain antibodies from camelid animals) as probes and identified a unique epitope on the SARS-CoV-2 spike protein with opposing functions across different SARS-CoV-2 variants. It enhances viral infection in pre-Omicron variants but inhibits it in the Omicron variant. We further investigated the molecular mechanism underlying these opposing effects. Although this epitope is not directly involved in receptor binding, nanobody binding to it modulates receptor binding and post-receptor-binding activities. This study marks the first discovery of a dual-function epitope on the SARS-CoV-2 spike protein that both enhances and inhibits viral infection across different viral variants. This discovery challenges the traditional categorization of epitopes, underscores the complex evolution of the SARS-CoV-2 spike protein, and offers new insights into antiviral antibody therapies.

## Introduction

Antibodies have long been used to investigate the structure and function of viral glycoproteins by targeting specific epitopes [1,2]. Typically, antibody binding to these epitopes either neutralizes the virus or has no impact on its ability to enter cells. However, recent research indicates that antibodies can enhance a virus’s entry into cells when they bind to certain epitopes, sparking interest in how they modulate the function of viral glycoproteins [3–7]. No epitopes have been identified that can both neutralize and enhance entry for different variants of the same virus. Discovering such dual-function epitopes would significantly advance our understanding of viral entry and evolution, underscoring the need for targeted antiviral therapies that consider epitope variability across viral variants.

**Fig 1:**
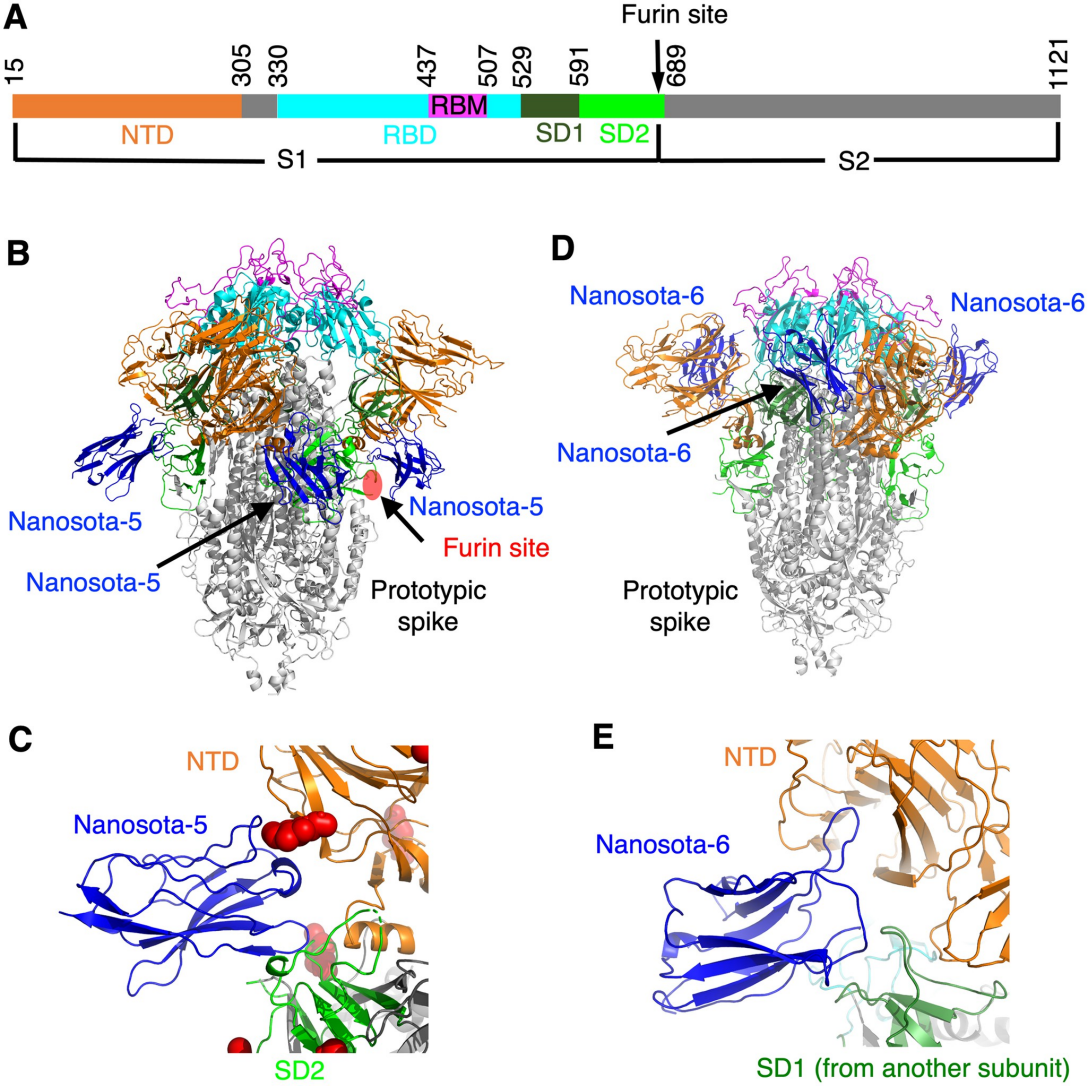
Structures of prototypic SARS-CoV-2 spike complexed with individual non-RBD-targeting nanobodies. **(A)** Schematic drawing of SARS-CoV-2 spike ectodomain (prototypic variant). S1: receptor-binding subunit. S2: membrane-fusion subunit. NTD: N-terminal domain of S1. RBD: receptor-binding domain of S1. RBM: receptor-binding motif of RBD. SD1: subdomain 1 of S1. SD2: subdomain 2 of S1. Furin site: cleavage site for the furin protease. **(B)** Structure of prototypic SARS-CoV-2 spike complexed with Nanosota-5. Three bound Nanosota-5 molecules are colored in blue. The spike domains are colored in the same way as (A). The furin cleavage site is labeled. **(C)** The binding site of Nanosota-5 on prototypic SARS-CoV-2 spike. A glycan N-linked to Asn61 on the spike is shown as red balls. NTD and SD2 bound by Nanosota-5 are from the same spike protomer. **(D)** Structure of prototypic SARS-CoV-2 spike complexed with Nanosota-6. Three bound Nanosota-6 molecules are colored in blue. **(E)** The binding site of Nanosota-6 on prototypic SARS-CoV-2 spike. NTD and SD1 bound by Nanosota-6 are from two spike protomers.

The spike protein of SARS-CoV-2 mediates viral entry into host cells [8–10]. The spike on the surface of mature virus particles is in the “pre-fusion” state. It is a homotrimer, consisting of three receptor-binding S1 subunits on top of a trimeric membrane-fusion S2 stalk. S1 contains a receptor-binding domain (RBD), an N-terminal domain (NTD), and two subdomains SD1 and SD2 (Fig 1A). The RBD exists in two conformations within the spike: standing up for receptor binding and lying down for immune evasion [11,12]. The RBD includes a core subdomain and a receptor-binding motif (RBM). During viral entry, the RBD binds to its host receptor ACE2, and the spike is cleaved at the S1/S2 boundary by a host protease (e.g., proprotein convertase furin, cell-surface TMPRSS2, or lysosomal cathepsins) [12–15]. Then S1 dissociates and S2 undergoes a dramatic structural change to fuse viral and host membranes [8–10]. After membrane fusion, S2 is in the “post-fusion” state. While the post-fusion spike is the most stable and lowest-energy state, the pre-fusion spike must overcome an initial energy barrier to transition into the post-fusion state [10].

The spike protein of SARS-CoV-2 is key to triggering antibody responses; it is also the sole antigen in many COVID-19 vaccines and the main target for antibody-based treatments [10,16]. Antibodies work by latching onto specific epitopes on the spike protein. Neutralizing antibodies block SARS-CoV-2 from entering cells in two main ways. First, by binding to the RBM epitopes on the spike, they prevent the virus from attaching to the ACE2 receptor [17–20]. Second, they can attach to non-RBD epitopes on the spike, keeping the spike in its pre-fusion state and preventing it from fusing membranes [21–23]. The ways that antibodies could enhance viral entry are less understood, but they may promote the RBD/ACE2 interaction by maintaining the RBD in the standing up position [3]. There is ongoing debate about whether such antibodies actually worsen SARS-CoV-2 infections *in vivo* [3]. Since neutralizing antibodies and entry-enhancing antibodies act in fundamentally different ways with completely opposite outcomes, it is unconceivable that a single antibody could both neutralize the virus and facilitate its entry into cells across various viral variants.

Nanobodies are the antigen-binding domain of heavy-chain-only antibodies produced by camelid animals [24–27]. Here we used nanobodies as probes to investigate the functions of epitopes on the SARS-CoV-2 spike. We identified an intriguing epitope, located outside the RBD of the spike, that enhances the cell entry of prototypic SARS-CoV-2, but neutralizes the cell entry of Omicron variant. We further examined the molecular mechanisms for such dual functions of this epitope. Our study has important implications for the structure, function and evolution of SARS-CoV-2 spike and for the efficacy and safety of antibody therapeutics.

## Results

### Discovery of three non-RBD epitopes on prototypic spike

In a recent study, we described the discovery of three RBD-targeting nanobodies, named Nanosota-2, Nanosota-3 and Nanosota-4, from an alpaca immunized with prototypic SARS-CoV-2 spike [28]. Here prototypic spike refers to the spike protein from the original SARS-CoV-2 variant plus an additional D614G mutation. Each of the three nanobodies blocks ACE2 from binding to the RBD and thereby neutralizes prototypic SARS-CoV-2 entry. In the current study, we discovered three more nanobodies, named Nanosota-5, Nanosota-6 and Nanosota-7, from the same alpaca immunized with prototypic spike. We determined the cryo-EM structures of prototypic spike complexed with either Nanosota-5 or Nanosota-6 (Figs 1B, 1C, 1D, 1E, S1 and S2 and S1 Table). The structures revealed that Nanosota-5 binds to the NTD and SD2 from the same spike protomer (Figs 1B, 1C and S1), whereas Nanosota-6 binds to the NTD and SD1 from two different spike protomer (Figs 1D, 1E and S2). The epitopes of Nanosota-5 and -6 are both located outside the RBD and do not overlap with the ACE2-binding region. The location of the Nanosota-7 epitope could not be identified using cryo-EM probably due to its flexibility, but ELISA indicated that the Nanosota-7 epitope is located outside the RBD but within S1 (S3 Fig). In sum, Nanosota-5, Nanosota-6 and Nanosota-7 all target non-RBD epitopes.

### Non-RBD epitopes enhance cell entry of prototypic SARS-CoV-2 pseudoviruses

**Fig 2:**
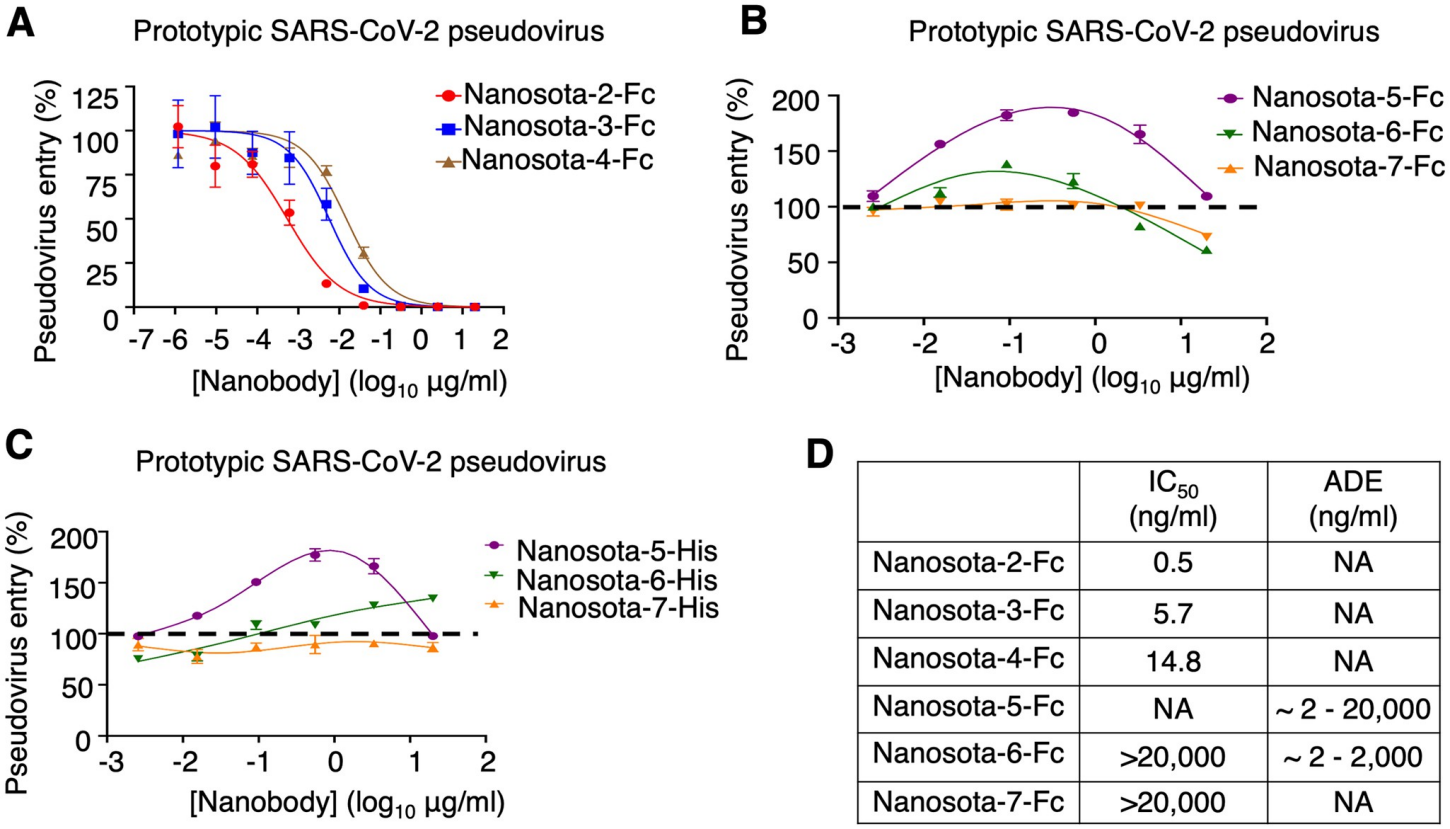
Non-RBD epitopes enhance the cell entry of prototypic SARS-CoV-2 pseudoviruses. Retroviruses pseudotyped with prototypic SARS-CoV-2 spike (i.e., prototypic SARS-CoV-2 pseudoviruses) entered ACE2-expressing cells in the presence of one of the nanobodies at various concentrations. Entry efficiency was characterized as the luciferase signal accompanying entry. The efficacy of each nanobody in neutralizing pseudovirus entry was expressed as the concentration capable of neutralizing pseudovirus entry by 50% (i.e., IC_50_). **(A)** Activities of three Fc-tagged RBD-targeting nanobodies: they all potently neutralized SARS-CoV-2 pseudovirus entry. Error bars represent SEM (n = 3). **(B)** Activities of three Fc-tagged non-RBD-targeting nanobodies: Nanosota-5-Fc enhances pseudovirus entry, Nanosota-6-Fc enhances pseudovirus entry at low concentrations and neutralizes pseudovirus entry at high concentrations, and Nanosota-7-Fc neutralizes pseudovirus entry at high concentrations. Error bars represent SEM (n = 4). **(C)** Activities of three His-tagged non-RBD-targeting nanobodies: Nanosota-5-His and Nanosota-6-His enhance pseudovirus entry using an FcR-independent mechanism. Error bars represent SEM (n = 4). **(D)** Summary of the activities of the six spike-binding nanobodies. NA: not available.

To investigate the functions of the newly identified non-RBD epitopes, we studied how nanobodies targeting these epitopes, Nanosota-5, -6 and -7, influence the cell entry of prototypic SARS-CoV-2 pseudoviruses. The three RBD-targeting nanobodies, Nanosota-2, -3 and -4 were used for comparison. To facilitate protein purification and biochemical studies, we introduced a C-terminal Fc tag to each of the nanobodies (named Nanosota-2-Fc, etc.). Subsequently, retroviruses pseudotyped with SARS-CoV-2 spike (i.e., SARS-CoV-2 pseudoviruses, etc.) were used to infect ACE2-expressing HEK293T cells in the presence of each of the Fc-tagged nanobodies. The results revealed that Nanosota-2-Fc, -3-Fc, and -4-Fc all potently neutralized SARS-CoV-2 pseudovirus entry (Fig 2A). However, Nanosota-6-Fc and -7-Fc only slightly neutralized SARS-CoV-2 pseudovirus entry at high concentrations (Fig 2B). Interestingly, Nanosota-5-Fc at a wide concentration range and Nanosota-6-Fc at lower concentrations significantly enhanced SARS-CoV-2 pseudovirus entry (Fig 2B). This resembled the antibody-dependent enhancement (ADE) of coronavirus entry that we observed previously using RBD-targeting neutralizing IgGs [4]. We previously showed that RBD-targeting IgGs, which contain an Fc tag, guide coronavirus entry into FcR-expressing cells [4]. However, Nanosota-5-Fc and -6-Fc enhanced SARS-CoV-2 pseudovirus entry into HEK293T cells that express ACE2, not FcR. To confirm that FcR did not play a role in the enhanced viral entry, we repeated the SARS-CoV-2 pseudovirus entry assay using His-tagged nanobodies (named Nanosota-5-His, etc.). Both Nanosota-5-His and -6-His enhanced SARS-CoV-2 pseudovirus entry despite having no Fc tag (Fig 2C). Therefore, two of the three non-RBD epitopes on prototypic spike enhance the cell entry of prototypic SARS-CoV-2 pseudoviruses through an FcR-independent mechanism (Fig 2D).

We selected Nanosota-5 for further investigation because it was the most effective at enhancing prototypic SARS-CoV-2 pseudovirus entry among the non-RBD-targeting nanobodies. Additional pseudovirus entry assays revealed that even at very high concentrations (e.g., 0.4 mg/ml), Nanosota-5-Fc continued to enhance the cell entry of prototypic SARS-CoV-2 pseudoviruses (S4A Fig). Moreover, Nanosota-5-Fc enhanced the cell entry of both the alpha and delta variants of SARS-CoV-2 pseudoviruses (S4B Fig). Thus, Nanosota-5 enhances the cell entry of prototypic and other pre-Omicron SARS-CoV-2 variants. Additionally, the distance between two Nanosota-5 epitopes on the prototypic trimeric spike is 143 Å, suggesting that Nanosota-5-Fc cannot bind two spike protomers in the same trimeric spike simultaneously. These results provided additional insights into Nanosota-5’s impact on the cell entry of pre-Omicron SARS-CoV-2 variants.

### A non-RBD epitope enhances cell infection of live prototypic SARS-CoV-2

**Fig 3:**
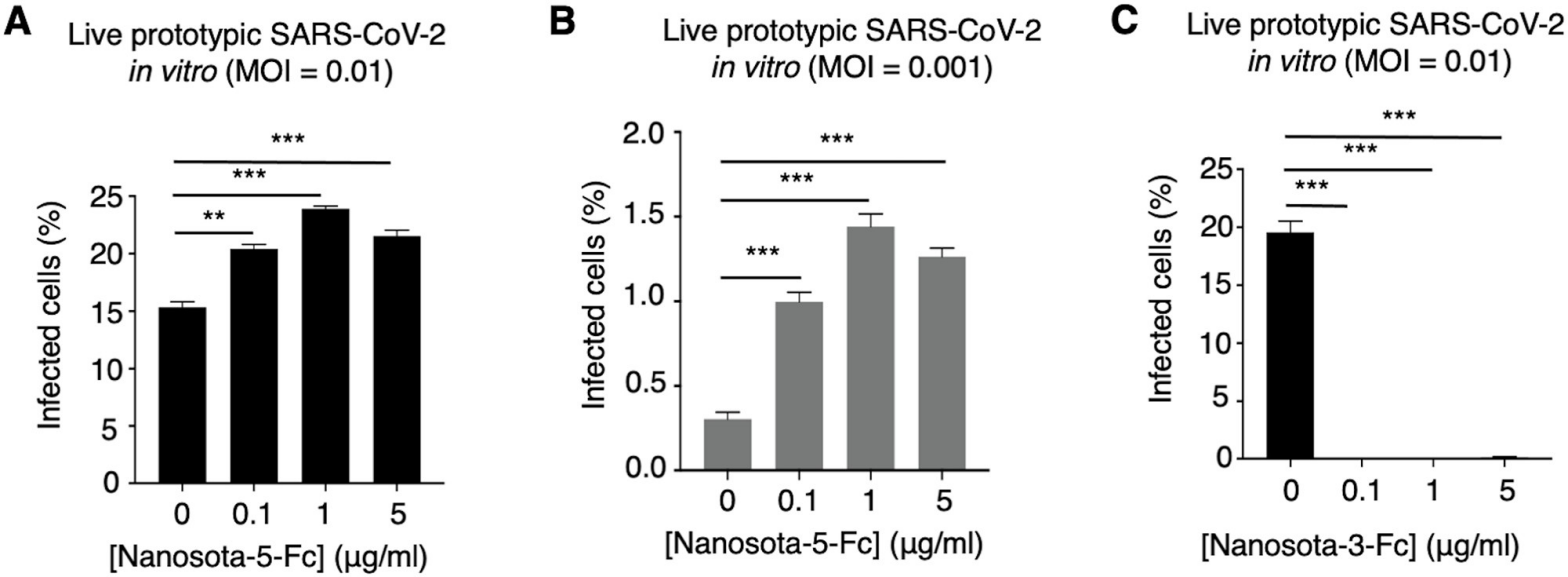
A non-RBD epitope enhances cell infection by live prototypic SARS-CoV-2. Recombinant SARS-CoV-2-Venus (prototypic variant) was used to infect Vero E6 cells in the presence of various concentrations of Nanosota-5-Fc. PBS buffer was used as a negative control. **(A)** Nanosota-5-Fc enhances cell infection by live prototypic SARS-CoV-2 at a high virus titer. **(B)** Nanosota-5-Fc enhances cell infection by live prototypic SARS-CoV-2 at a low virus titer. **(C)** Nanosota-3-Fc, an RBD-targeting neutralizing nanobody, was used for comparison to Nanosota-5-Fc. Infection efficiency was characterized as the percentage of infected cells detected by flow cytometry. MOI stands for multiplicity of infection. Comparisons of viral infections between the negative control and various concentrations of Nanosota-5-Fc or Nanosota-3-Fc were performed using an unpaired two-tailed Student’s *t*-test. Error bars represent SEM (n = 3). ***p<0.001; **p<0.01.

We further explored whether non-RBD epitopes can enhance the infection of live prototypic SARS-CoV-2 in cultured cells. Specifically, we examined the infection efficiency of live SARS-CoV-2 in ACE2-expressing Vero cells in the presence or absence of Nanosota-5-Fc. Two different virus titers and three different nanobody concentrations were tested. At all nanobody concentrations, significantly enhanced viral infection was observed at the high virus titer (Fig 3A) and became even more prominent at the low virus titer (Fig 3B). As a comparison, Nanosota-3-Fc, which targets the RBD, potently neutralized the infection of live prototypic SARS-CoV-2 (Fig 3C). Thus, the Nanosota-5 epitope not only enhances the cell entry of prototypic SARS-CoV-2 pseudoviruses, but also enhances the cell infection of live prototypic SARS-CoV-2.

### The same non-RBD epitope neutralizes SARS-CoV-2 Omicron variant

**Fig 4:**
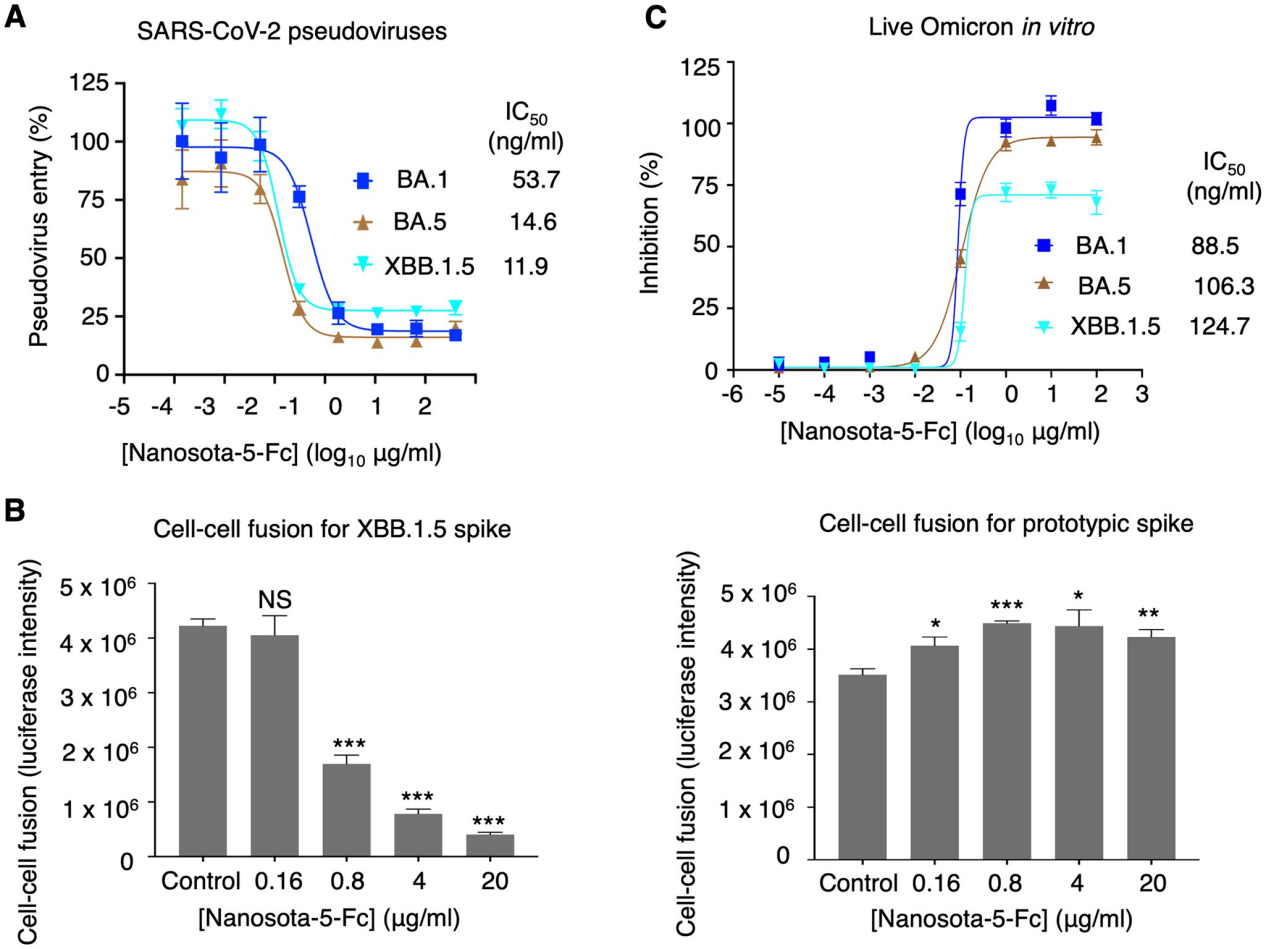
A non-RBD epitope neutralizes the cell entry of SARS-CoV-2 Omicron variant. **(A)** Pseudovirus entry assay shows that Nanosota-5-Fc neutralizes the cell entry of three Omicron subvariants. Error bars represent SEM (n = 4). IC_50_ values were calculated for each of the three Omicron subvariants. **(B)** Cell-cell fusion assay shows that Nanosota-5-Fc decreases XBB.1.5-spike-mediated cell-cell fusion (left), but increases prototypic-spike-mediated cell-cell fusion (right). Spike-expressing cells and ACE2-expressing cells were incubated together for fusion in the presence of various concentrations of Nanosota-5-Fc. Comparisons of cell-cell fusion between the negative control (PBS buffer) and various concentrations of Nanosota-5-Fc were performed using an unpaired two-tailed Student’s *t*-test. Error bars represent SEM (n = 4). ***p<0.001; **p<0.01. **p<0.05. NS: not statistically significant. **(C)** Nanosota-5-Fc neutralizes cell infection by live Omicron subvariants. The efficacy of Nanosota-5-Fc against Omicron subvariants was calculated and expressed as the concentration capable of maintaining the cell viability by 50% (IC_50_) compared to control virus. Error bars represent SEM (n = 4).

After studying the function of non-RBD epitopes in the cell entry of prototypic SARS-CoV-2, we further investigated their function in the cell entry of SARS-CoV-2 Omicron variant. To this end, SARS-CoV-2 Omicron pseudoviruses were used to enter ACE2-expressing HEK293T cells in the presence of Nanosota-5-Fc (Fig 4A). The pseudoviruses of three different Omicron subvariants were tested, including the early subvariant BA.1, the later subvariant BA.5 and the recent subvariant XBB.1.5. Surprisingly, Nanosota-5-Fc effectively neutralized the pseudovirus entry of all three Omicron subvariants (Fig 4A). To validate the above result, we performed a cell-cell fusion assay where cells expressing the spike protein (from either prototypic or XBB.1.5 SARS-CoV-2) and cells expressing ACE2 were incubated together for fusion in the presence of Nanosota-5-Fc. The result revealed that while Nanosota-5 enhanced the cell-cell fusion for prototypic spike, it inhibited the cell-cell fusion for XBB.1.5 spike (Fig 4B). We also repeated the live SARS-CoV-2 infection assay in the presence of Nanosota-5-Fc, this time using live BA.1, BA.5, and XBB.1.5 instead of prototypic SARS-CoV-2. The result demonstrated that Nanosota-5-Fc neutralized all three live Omicron subvariants in cultured cells (Fig 4C). Together, all these lines of evidence reveal that the Nanosota-5 epitope has opposing effects on the functions of the spike proteins from prototypic SARS-CoV-2 and Omicron variant: entry-enhancing for the former and neutralizing for the latter.

**Fig 5:**
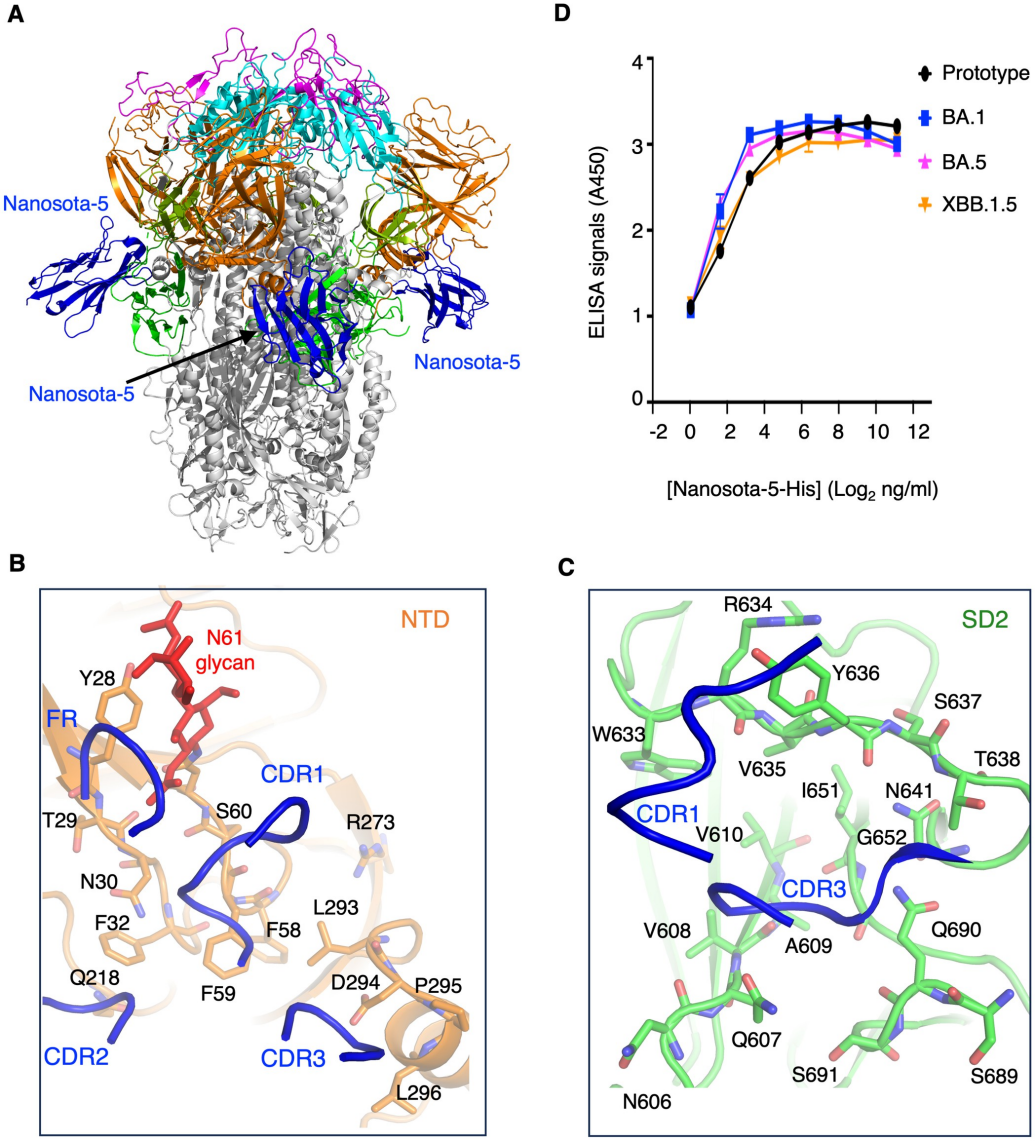
Structure of XBB.1.5 spike complexed with non-RBD-targeting nanobody. **(A)** Structure of XBB.1.5 spike complexed with Nanosota-5. Three bound Nanosota-5 molecules are colored in blue. The spike domains are colored as in Fig 1A. **(B)** Interactions between Nanosota-5 and the NTD of XBB.1.5 spike. Three complementarity-determining regions (CDRs) and part of a framework region (FR) of Nanosota-5 (shown as ribbons) are directly involved in binding the NTD residues (shown as sticks). **(C)** Interactions between Nanosota-5 and the SD2 of XBB.1.5 spike. CDR1 and CDR3 of Nanosota-5 (shown as ribbons) are directly involved in binding the SD2 residues (shown as sticks). All Nanosota-5-contacting residues in the NTD and SD2 are conserved from the prototypic to Omicron spikes (see S6 Fig). (D) ELISA comparing the binding of Nanosota-5-His to the spike ectodomains from prototypic SARS-CoV-2, Omicron BA.1, Omicron BA.5, and Omicron XBB.1.5.

To understand how Nanosota-5 binds to XBB.1.5 spike, we determined the cryo-EM structure of XBB.1.5 spike complexed with Nanosota-5 (Figs 5A and S5 and S1 Table). The result showed that Nanosota-5 binds to the same epitope on XBB.1.5 spike as it does prototypic spike. Again, this epitope is located at the junction of the NTD and SD2 from the same spike protomer. Nanobodies contain three complementarity-determining regions (CDRs) and four framework regions (FRs). All three CDRs and a part of an FR of Nanosota-5 are involved in contacting the NTD (Fig 5B), while its CDR1 and CDR3 are involved in contacting the SD2 (Fig 5C). Surprisingly, all of the Nanosota-5-contacting residues are conserved between prototypic and Omicron spikes, including a glycan N-linked to Asn61 in prototypic spike (corresponding to Asn58 in XBB.1.5 spike) (Figs 5B, 5C and S6). Additionally, we examined the residues outside but near the Nanosota-5-binding site and identified a few residue differences between prototypic and XBB.1.5 spikes. We found that these differences did not significantly affect the surface electrostatic potential in the neighboring regions of the Nanosota-5-binding site (S7 Fig). Indeed, ELISA results showed that Nanosota-5-His exhibited similar binding affinities for the recombinant spike ectodomains from prototypic SARS-CoV-2 and three Omicron subvariants (BA.1, BA.5, and XBB.1.5) (Fig 5D). Thus, the Nanosota-5 epitope on the spikes from different SARS-CoV-2 variants is conserved, suggesting that its varying effects on the functions of spikes from different viral variants are due to structural differences between prototypic and Omicron spikes outside of this epitope.

### Molecular mechanism for the dual function of the non-RBD epitope

**Fig 6:**
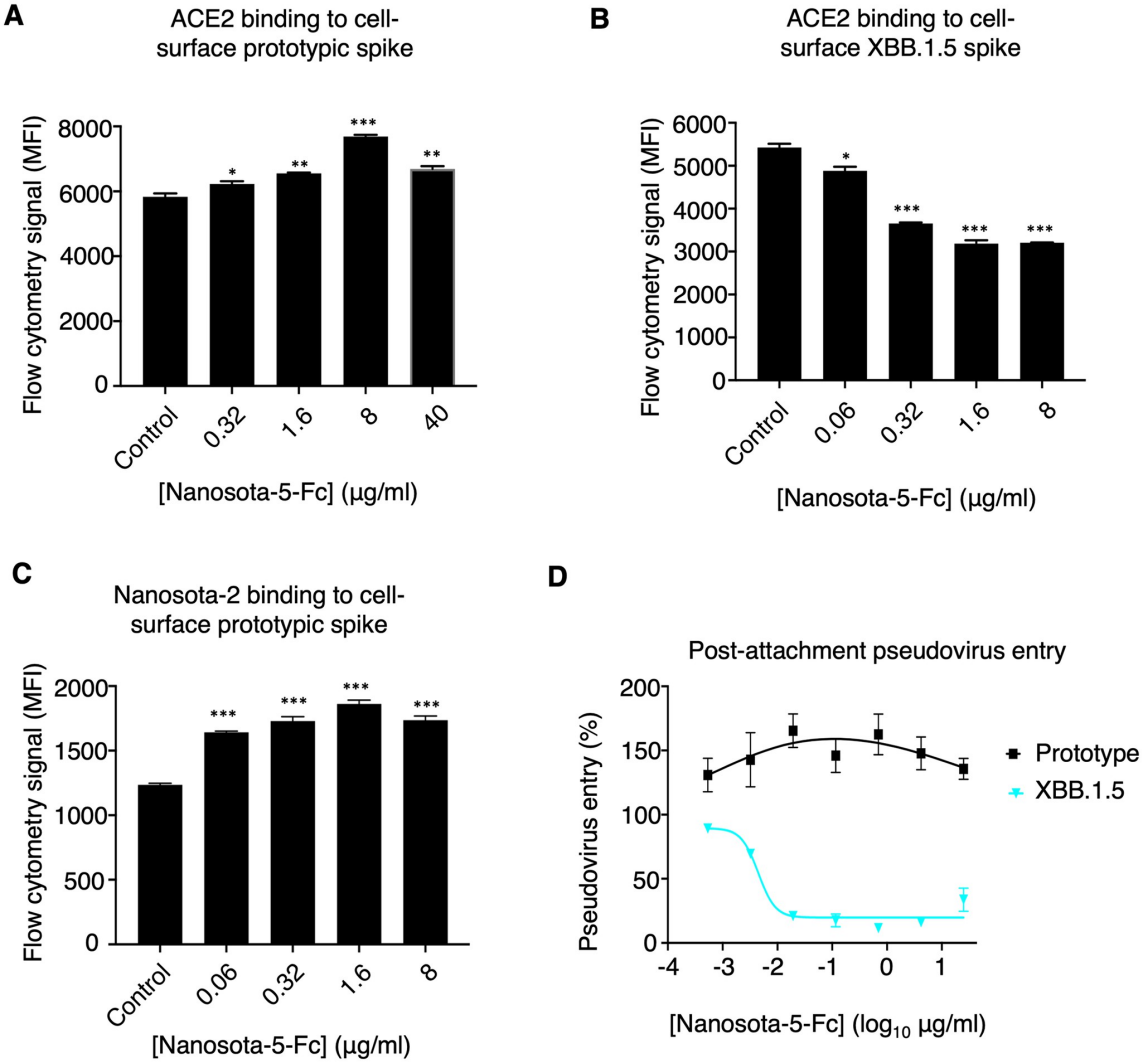
Mechanism for non-RBD epitopes exhibiting opposite functions across different SARS-CoV-2 variants. Flow cytometry assay shows that Nanosota-5-Fc increases ACE2 binding by prototypic spike **(A)** but decreases ACE2 binding by XBB.1.5 spike **(B)**. A mixture of recombinant ACE2 ectodomain and recombinant Nanosota-5-Fc was incubated with spike-expressing cells, and the binding between the recombinant ACE2 ectodomain and the cell-surface spike was measured using flow cytometry. Nanosota-2, which, like ACE2, only binds to the standing up RBD, replaced the ACE2 ectodomain in **(C)**. PBS buffer was used as a negative control. Comparisons between the negative control and Nanosota-5-Fc for their impact on spike/ACE2 binding were performed using an unpaired two-tailed Student’s t-test. Error bars represent SEM (n = 3). ***p<0.001, **p<0.01, *p<0.05. **(D)** Post-attachment pseudovirus entry. Pseudoviruses were incubated with cells before adding Nanosota-5-Fc and allowing pseudovirus entry to occur.

We investigated the molecular mechanism behind the non-RBD epitope having opposing effects on the functions of spikes from different SARS-CoV-2 variants. We examined how Nanosota-5-Fc affects the binding interactions between the SARS-CoV-2 spike and ACE2. To do this, recombinant ACE2 was incubated with cell-surface spikes in the presence or absence of Nanosota-5-Fc, and the interaction between the spikes and ACE2 was measured using flow cytometry. Both prototypic and XBB.1.5 spikes were tested. The results showed that Nanosota-5-Fc increased the binding affinity between prototypic spike and ACE2 (Fig 6A), while decreasing the binding affinity between XBB.1.5 spike and ACE2 (Fig 6B). Since only the standing-up RBD can bind to ACE2, these results suggest that Nanosota-5-Fc promotes more RBDs to stand up in prototypic spike, while having the opposite effect on XBB.1.5 spike. To confirm this conclusion, we further investigated how Nanosota-5-Fc affects the binding interaction between prototypic spike and Nanosota-2, which, like ACE2, is only accessible to the standing-up RBD [28]. Flow cytometry results confirmed that Nanosota-5-Fc increased the binding affinity between prototypic spike and Nanosota-2 (Fig 6C). Therefore, the Nanosota-5 epitope has opposing effects on the ACE2-binding affinity of prototypic and XBB.1.5 spikes, likely by promoting the RBD to stand up.

We also analyzed whether Nanosota-5 affects post-attachment events during the viral entry process. To do this, we incubated pseudoviruses with cells before adding Nanosota-5-Fc and allowing pseudovirus entry to occur. The results showed that even after viral attachment to cells had occurred, Nanosota-5-Fc still enhanced the cell entry of prototypic pseudoviruses, while neutralizing the cell entry of XBB.1.5 pseudoviruses (Fig 6D). Therefore, Nanosota-5 has opposing effects not only on ACE2 binding between prototypic and XBB.1.5 spikes but also on post-attachment steps between prototypic and XBB.1.5 pseudoviruses.

Since the Nanosota-5 epitope is near the furin cleavage site (Fig 1B), we examined whether the furin cleavage site impacts Nanosota-5’s binding to the SARS-CoV-2 spike. ELISA results showed that Nanosota-5-His had similar binding affinities for prototypic spike, regardless of the presence of the furin cleavage site (S8A Fig). Next, we investigated whether Nanosota-5 affects the furin cleavage of the spikes. To do this, we co-expressed Nanosota-5-Fc with either prototypic or XBB.1.5 spike in cells. The results suggested that Nanosota-5-Fc inhibited furin cleavage of both prototypic and XBB.1.5 spikes (S8B Fig). It is important to note that during molecular maturation, the majority of the spike molecules had already been cleaved (S8B Fig). Therefore, even though Nanosota-5 inhibits furin cleavage of the spikes, it may not have a significant impact on viral entry through furin cleavage inhibition. Additionally, since Nanosota-5 inhibits furin cleavage of both prototypic and XBB.1.5 spikes, and this study focuses on the opposing effects of Nanosota-5 on these spikes, Nanosota-5’s inhibition of furin cleavage is not a primary focus of the current study.

Since pre-Omicron and Omicron spikes differ in their sensitivity to TMPRSS2 [29], we analyzed whether TMPRSS2 affects Nanosota-5’s impact on SARS-CoV-2 entry. Pseudovirus entry assay results showed that in the presence of TMPRSS2, Nanosota-5-Fc still enhanced the entry of prototypic pseudoviruses while neutralizing the entry of Omicron pseudoviruses into cells overexpressing TMPRSS2 (S8C Fig). Therefore, the opposing effects of Nanosota-5 on prototypic and Omicron spikes are not due to the different TMPRSS2 sensitivities between the two spikes.

## Discussion

Epitopes on viral glycoproteins are not just tools for deciphering the structure and function of these molecules; they are also fundamental to developing antiviral vaccines and antibody-based treatments. Typically, epitopes are classified as either neutralizing, non-neutralizing, or infection-enhancing. However, by using nanobodies in our research, we have identified a SARS-CoV-2 spike epitope that has a dual role: it neutralizes one variant of SARS-CoV-2 and enhances infection in another. This discovery challenges the traditional categorization of epitopes, underscores the complex evolution of SARS-CoV-2 spike, and offers new insights into antiviral antibody therapies.

Infection-enhancing epitopes in coronavirus spikes have attracted great scientific interest. The classical pathway for coronavirus entry into cells relies on host receptors, where the viruses attach to their receptor, undergo endocytosis, and subsequently fuse viral and host membranes [10]. Previously, we discovered a unique molecular mechanism for ADE of coronavirus entry that is dependent on FcR but independent of host receptors. Specifically, neutralizing antibodies that target the RBD simultaneously bind to the RBD on the virus and FcR on the cell surfaces. This interaction guides coronaviruses into cells expressing FcR, such as macrophages (S9 Fig) [4]. This mechanism has later been validated *in vivo* [30–33]. More recently, a group of human antibodies, targeting a non-RBD epitope, was isolated from COVID-19 patients and found to enhance SARS-CoV-2 infection by promoting the spike’s binding to its ACE2 receptor [3]. While resembling ADE, this mechanism has not yet been confirmed *in vivo*. Additionally, the infection-enhancing activity of these human antibodies requires Fc, although the reason for this remains unclear [3]. In our current study, we identified two entry-enhancing epitopes outside the RBD of prototypic SARS-CoV-2 spike and delved deeper into one of them, recognized by Nanosota-5. This epitope is situated at the junction between the NTD and SD2. It partially overlaps with the entry-enhancing epitope mentioned earlier, which is recognized by human antibodies (S10 Fig). In contrast to the FcR-dependent ADE observed with the RBD epitope, the Nanosota-5 epitope enhances entry through an FcR-independent mechanism. It is not clear whether the Nanosota-5 epitope causes ADE *in vivo*, which will be investigated in future studies. Overall, our research offers new perspectives on how non-RBD epitopes can increase prototypic SARS-CoV-2’s ability to enter cells *in vitro*.

Remarkably, the Nanosota-5 epitope exhibits dual functions: it enhances the cell entry of prototypic SARS-CoV-2 but neutralizes the cell entry of Omicron variant, including BA.1, BA.5, and XBB.1.5 subvariants. This discovery challenges conventional epitope paradigms. To decipher the molecular mechanisms underpinning how the same non-RBD epitope can exert opposing effects on different SARS-CoV-2 variants, we conducted comparative studies between prototypic and Omicron spikes. Our investigations revealed that for prototypic spike, Nanosota-5-Fc promotes its ACE2 binding, thereby enhancing viral entry, and also increases its post-attachment activity, likely facilitating its transition to the post-fusion structure. Surprisingly, although our flow cytometry data showed that Nanosota-5 promotes the prototypic spike to bind ACE2, our cryo-EM study of the prototypic spike in complex with Nanosota-5 revealed that all Nanosota-5-bound prototypic spike molecules were in the three-RBD down conformation, which disfavors ACE2 binding. This contrasts with the cryo-EM studies on unliganded prototypic spike, which typically have 40–50% of the molecules with at least one RBD in the standing up position [11,12]. The reason for the discrepancy between our flow cytometry and cryo-EM results is unclear but could be due to the cryo-EM procedure (for instance, Nanosota-5-bound open spike might be too unstable to be characterized by cryo-EM). The open and closed populations of Nanosota-5-bound prototypic spike will be further investigated in future cryo-EM studies. Nevertheless, using flow cytometry, we also demonstrated that Nanosota-5 promotes the prototypic spike to bind Nanosota-2, which, like ACE2, only binds to the standing up RBD of the spike [28]. Overall, our data suggest that Nanosota-5 induces the RBD to stand up in prototypic spike, facilitating ACE2 or Nanosota-2 binding. It has been shown that the standing up RBD destabilizes the pre-fusion spike, allowing it to transition more easily to its post-fusion structure [10,34], which aligns with our post-attachment entry data for prototypic pseudoviruses. In contrast, Nanosota-5 reduces ACE2 binding by Omicron spike and inhibits the post-attachment entry of Omicron pseudoviruses, likely by inducing the RBDs to adopt the lying down conformation in Omicron spike. More detailed mechanistic analysis of the opposing effects observed with the Nanosota-5 epitope across different SARS-CoV-2 variants will be conducted in future studies.

Our study sheds light on the potential for more targeted antiviral antibody therapies. We have found that certain epitopes on viral glycoproteins can have dual roles: they might help one virus variant enter cells but block another variant. This discovery highlights the complexity of viral evolution; accordingly, our approaches to antiviral antibody therapies need to consider the versatile nature of epitopes on viral glycoproteins.

## Methods

### Cell lines, plasmids and virus

HEK293T cells and Calu-3 cells (American Type Culture Collection (ATCC)) were grown in Dulbecco’s modified Eagle medium (DMEM) (containing 10% fetal bovine serum, 2 mM L-glutamine, 100 units/mL penicillin, and 100 μg/mL streptomycin). 293F cells (ThermoFisher) were grown in FreeStyle 293 Expression Medium (ThermoFisher). Vero E6 cells (ATCC) were cultured in Eagle’s minimal essential medium (EMEM) (containing 100 units/ml penicillin, 100 μg/ml streptomycin, and 10% fetal bovine serum). ss320 *E*. *coli* (Lucigen) and TG1 *E*. *coli* (Lucigen) were grown in 2YT medium. All mammalian cells were authenticated by ATCC using STR profiling and were also tested for mycoplasma contamination. No commonly misidentified cell lines were used.

Original SARS-CoV-2 spike gene (GenBank: QHD43416.1) and human TMPRSS2 gene (UniProt: O15393) were synthesized (GenScript). Mutations were introduced to the original SARS-CoV-2 spike gene to generate the prototypic SARS-CoV-2 spike gene (encoding the spike protein from Wuhan variant plus D614G mutation), alpha variant (GISAID: EPI_ISL_6135157), delta variant (GenBank: UEM53021.1), Omicron BA.1 subvariant (GISAID: EPI_ISL_6590782.2), Omicron BA.5 subvariant (GISAID: EPI_ISL_12954165), and Omicron XBB.1.5 subvariant (GISAID: EPI_ISL_17774216). Each of the spike genes was cloned into the pcDNA3.1(+) vector.

Genes encoding spike ectodomain (residues 14–1211), RBD (residues 319–529), and S1 (residues 14–685) from prototypic SARS-CoV-2 were each subcloned into Lenti-CMV vector (Vigene Biosciences) with an N-terminal tissue plasminogen activator (tPA) signal peptide and a C-terminal His tag. For the spike ectodomain construct, a D614G mutation, two mutations in the furin cleavage site (from RRAR to AGAR), and six proline mutations were introduced to the S2 subunit region to stabilize the spike protein in its prefusion state [35,36]. Plasmids encoding Fc-tagged Nanosota-5, -6, and -7 were constructed in the same way as above except that a C-terminal human IgG_1_ Fc tag replaced the His tag.

Genes encoding monomeric Nanosota-5, -6, and -7 were each cloned into PADL22c vector (Antibody Design Labs) with an N-terminal PelB leader sequence and C-terminal His tag and HA tag.

The BAC cDNA clones of recombinant SARS-CoV-2 were kindly provided by Dr. Luis Enjuanes. Recombinant SARS CoV-2-Venus (rSARS-CoV-2-Venus) BAC was constructed as previously described [37]. The SARS-CoV-2_MA30_ virus was described previously [38]. The Omicron viruses (hCoV-19/USA/GA-EHC-2811C/2021 for BA.1, hCoV-19/USA/COR-22-063113/2022 for BA.5, and hCoV-19/USA/MD-HP40900/2022 for XBB.1.5) were obtained through BEI Resources, NIH. Experiments involving live infectious SARS-CoV-2 were conducted at the University of Iowa and the University of Louisville in approved biosafety level 3 laboratories.

### Screening of nanobody phage display library

The same nanobody phage display library that was recently used to screen for RBD-targeting nanobodies was also used in the current study to screen for non-RBD-targeting nanobodies [28]. Briefly, 5 μg purified SARS-CoV-2 spike ectodomain coated on an ELISA plate was used for one round of bio-panning. 100 μl phages from the phage library were added to the coated spike and incubated for 1 hour. After washing, the retained phages were eluted and then used to infect ss320 *E*. *coli*. The infected ss320 *E*. *coli* were spread onto 2YT AG plates, and single colonies were picked and induced by 1 mM IPTG to express individual nanobodies. The supernatants were subjected to ELISA for identification of strong binders against prototypic SARS-CoV-2 spike.

### Protein expression and purification

Nanosota-5, -6 and -7 (with a His tag) were purified as previously described [39]. Briefly, nanobody expression was induced by 1 mM IPTG and purified from the periplasm of ss320 *E*. *coli*. The pellets were collected and re-suspended in 15 ml TES buffer (0.2 M Tris pH 8, 0.5 mM EDTA, 0.5 M sucrose), shaken on ice for 1 hour, diluted with 40 ml ¼ TES buffer, and then shaken on ice for another hour. The proteins in the supernatant were sequentially purified using an Ni-NTA column and a Superdex200 gel filtration column (Cytiva).

SARS-CoV-2 spike ectodomain (with a His tag), RBD (with a His tag) and individual Fc-tagged nanobodies were prepared from 293F cells as previously described [40]. Briefly, lentiviral particles were packaged using the plasmid encoding one of the above proteins and then used to infect 293F cells for selection of stable cell lines in the presence of Puromycin (Gibco). The proteins were harvested from the supernatant of respective cell culture medium, purified on Ni-NTA column for His-tagged proteins or on Protein A column for Fc-tagged proteins, and purified further on Superdex200 gel filtration column (Cytiva).

To prepare the complexes of the spike and individual nanobodies, 2 mg SARS-CoV-2 spike and each of the His-tagged nanobodies (with the nanobody in excess) were incubated at room temperature for 30 minutes. The above samples were then subjected to gel filtration using a Superose 6 increase 10/300 GL column (Cytiva).

### ELISA

To detect the binding between SARS-CoV-2 spike ectodomain and HA-tagged nanobodies from the supernatant of ss320 *E*. *coli*, ELISA was conducted as previously described [39]. Briefly, ELISA plates were coated with purified SARS-CoV-2 spike ectodomain and were then incubated sequentially with the supernatant of ss320 *E*. *coli* (containing nanobodies) and HRP-conjugated anti-HA antibody (1:5,000) (Sigma). ELISA substrate (Invitrogen) was added and the reactions were stopped using 1N H_2_SO_4_. The absorbance at 450 nm (A_450_) was measured using a Synergy LX Multi-Mode Reader (BioTek).

To detect the binding between SARS-CoV-2 spike domains and purified HA-tagged nanobodies, ELISA plates were coated with SARS-CoV-2 spike domains and were then incubated sequentially with the HA-tagged nanobodies and HRP-conjugated anti-HA antibody (1:5,000) (Sigma). The remaining procedure was the same as above.

### Pseudovirus entry assay

The activities of nanobodies in SARS-CoV-2 entry were evaluated using pseudovirus entry assay as previously described [40]. Briefly, to prepare the pseudoviruses, HEK293T cells were co-transfected with a pcDNA3.1(+) plasmid encoding SARS-CoV-2 spike, a helper plasmid psPAX2 and a reporter plasmid plenti-CMV-luc. Pseudoviruses were collected 72 hours post-transfection, incubated with individual nanobody at different concentrations at 37°C for 1 hour, and then used to enter HEK293T cells stably expressing human ACE2 (HEK293T/hACE2 cells). After another 60 hours, cells were lysed. Aliquots of cell lysates were transferred to new plates, a luciferase substrate was added, and relative light units (RLUs) were measured using an EnSpire plate reader (PerkinElmer). The neutralization potency of each nanobody was calculated and expressed as the concentration of the nanobody capable of inhibiting pseudovirus entry by 50% (IC_50_).

To assess the effects of Nanosota-5-Fc on pseudovirus entry into TMPRSS2-expressing cells, HEK293T cells transiently expressing human ACE2 and TMPRSS2 were used instead of HEK293T/hACE2 cells. All other procedures remained the same as described above.

For the post-attachment pseudovirus entry assay, pseudoviruses were adsorbed onto HEK293T/hACE2 cells at 4°C for 1 hour. Unbound pseudoviruses were removed, and the cells were washed three times with cold DMEM. Serially diluted Nanosota-5-Fc was then added to the cells and incubated at 4°C for 1 hour. The plates were subsequently transferred to 37°C to allow viral entry. Pseudovirus entry results were measured in the same manner as described above.

### Construction of BAC cDNA clone of rSARS-CoV-2-Venus and virus rescue

Recombinant SARS-CoV-2-Venus (rSARS-CoV-2-Venus) was engineered using a GalK/kanamycin dual marker cassette as previously described [41]. The BAC cDNA clone of rSARS-CoV-2-Venus was analyzed using restriction enzyme digestion, PCR, and direct sequencing and shown to be correct. The BAC-SARS-CoV-2 cDNA clone was constructed as previously described [41].

Confluent monolayers of Vero E6 cells were transfected with 2.0 μg per well of rSARS-CoV-2-Venus BAC cDNA using Lipofectamine 3000. At 72 hours post transfection, cell supernatants were harvested, labeled as P0, and stored at -80°C. The P0 virus was used to infect fresh Calu3 cells to generate P1 stocks. P1 viral stocks were aliquoted, titered and stored at −80°C until use.

### Live SARS-CoV-2 infection in vitro

Cell infection of live prototypic SARS-CoV-2 in cultured cells by Nanosota-5-Fc was detected using flow cytometry. Briefly, rSARS-CoV-2-Venus was incubated with different concentrations of Nanosota-5-Fc in 200 ml DMEM at 37°C for 1 hour. Vero E6 cells (106 cells per well, 12-well plate, triplicates) were either mock inoculated or inoculated with the virus/nanobody mixture. After incubation at 37°C for 1 hour with gentle rocking every 15 minutes, the inocula were removed and the plates were overlaid with 10% FBS. 24 hours post-inoculation, cells were treated with 100 ml of trypsin for 2 minutes. Following this, 500 ml 10% FBS was added to terminate the trypsinization, and cell suspension was centrifuged with 1000 rpm/min for 5 minutes. Cell pellets were resuspended using Cytofix (BD Bioscience) and fixed for 30 minutes. Expression of Venus was evaluated via flow cytometry. All flow cytometry data were acquired using a BD FACSVerse and analysed with FlowJo software. A previously discovered RBD-targeting nanobody Nanosota-3-Fc was used as a comparison [28].

The neutralization potency of Nanosota-5-Fc against live Omicron infections was carried out as previously described [42]. Briefly, Nanosota-5-Fc was 10-times serially diluted in 50 μl virus growth medium and then was mixed with 50 μl of one of the live Omicron viruses (subvariants BA.1, BA.5 or XBB.1.5) (3000 TCID_50_/ml) at 37°C for an hour. The mixture was added to Vero E6 cells, which over express TMPRSS2, in 96-well plates and incubated at 37°C in 5% CO_2_ for 4 days. Cell viability was measured using a neutral red assay (Sigma-Aldrich). The efficacy of Nanosota-5-Fc against the Omicron viruses was calculated and expressed as the concentration capable of maintaining the cell viability by 50% (i.e., IC_50_) compared to the control virus.

### Cell-cell fusion assay

Cell-cell fusion was performed as described previously [43]. Briefly, HEK293T cells were co-transfected with the plasmid pFR-Luc (containing a luciferase gene whose expression is controlled by a synthetic promoter) and the plasmid expressing SARS-CoV-2 spike. Besides, HEK293T cells expressing ACE2 were transfected with the plasmid pBD-NF-κB (encoding a fusion protein that can activate the luciferase gene expression of the pFR-Luc). After the cells were cultured for 36 hours, Nanosota-5-Fc at various concentrations were added to the spike-expressing cells and incubated for 30 minutes. Subsequently, ACE2-expressing cells were overlaid onto spike-expressing cells. The luciferase gene expression was activated when cell-cell fusion occurred. After incubation for 6 hours, the cells were lysed, and relative luciferase units were measured using an EnSpire plate reader (PerkinElmer Life Sciences).

### Flow cytometry assay

To assess the effects of Nanosota-5-Fc on the binding between cell-surface spike and recombinant ACE2 ectodomain, various concentrations of Nanosota-5-Fc were incubated with spike-expressing HEK293T cells for 20 minutes. Then, 2 μg/ml human ACE2 ectodomain (containing a C-terminal His tag) was added and incubated with the cell and Nanosota-5-Fc mixture for another 20 minutes. Spike-bound ACE2 molecules were stained with a PE-conjugated anti-His-tag antibody and analyzed using flow cytometry. The results were analyzed using FlowJo software (version 10).

To assess the effects of Nanosota-5-Fc on the binding between cell-surface spike and recombinant Nanosota-2-His (which only binds to the standing up RBD in the spike), 0.5 μg/ml Nanosota-2-His replaced 2 μg/ml human ACE2 ectodomain in the above experiment.

### Furin cleavage inhibition assay

HEK293T cells were seeded into a 6-well plate and transfected with 1 μg of prototypic or XBB.1.5 spike-expressing plasmid. Different amounts (0, 0.2, 1, and 5 μg) of plasmids expressing Nanosota-5-Fc were co-transfected with the spike-expressing plasmids. Twenty-four hours post-transfection, cells were collected, and the cell surface spike (containing a C-terminal C9 tag) was detected using an anti-C9 antibody by Western blot.

### Cryo-EM grid preparation and data acquisition

4 μl purified complexes of SARS-CoV-2 spike ectodomain and nanobodies (~2.6 μM for prototypic spike/Nanosota-5, ~3.1 μM for prototypic spike/Nanosota-6, and ~2.4 μM for XBB.1.5 spike/ Nanosota-5) were supplemented with 8 mM CHAPSO immediately before grid preparation. Each complex was then applied to freshly glow-discharged Quantifoil R1.2/1.3 300-mesh copper grids (EM Sciences) and blotted for 4 seconds at 22°C under 100% chamber humidity and plunge-frozen in liquid ethane using a Vitrobot Mark IV (FEI). Cryo-EM data were collected using Latitude-S (Gatan) equipped with a K3 direct electron detector and with a Biocontinuum energy filter (Gatan). For the prototypic spike/Nanosota-5 and prototypic spike/Nanosota-6 complexes, the movies were collected at a nominal magnification of 130,000x (corresponding to 0.664 Å per pixel). For the XBB.1.5 spike/ Nanosota-5 complex, the movies were collected at a nominal magnification of 81,000x (corresponding to 1.1 Å per pixel). Statistics of cryo-EM data collection are summarized in S1 Table.

### Cryo-EM data processing, model building and refinement

Cryo-EM data were processed using cryoSPARC v3.3.2 [44], and the procedure is outlined in S1, S2 and S5 Figs. Briefly, dose-fractionated movies were subjected to Patch motion correction with MotionCor2 [45] and Patch CTF estimation with CTFFIND-4.1.13 [46]. Particles were then picked using both Blob picker and Template picker in cryoSPARC v3.3.2 and subjected to the Remove Duplicate Particles Tool. Junk particles were removed through multiple rounds of 2D classifications. Particles from the good 2D classes were used for Ab-initio Reconstruction of three or four maps. The initial models were set as the starting references for heterogeneous refinement (3D classification). The good 3D classes were then subjected to further homogeneous, non-uniform and CTF refinements to generate the final maps with applied C3 symmetry. Map resolutions were determined by gold-standard Fourier shell correlation (FSC) at 0.143 between the two half-maps. Local resolution variation was estimated from the two half-maps in cryoSPARC v3.3.2 or v4.0.3.

Initial model building of the spike/nanobody complexes was performed in Coot-0.8.9 [47] using PDB 7TGY and 8IOS as the starting model for prototypic and XBB.1.5 spikes, respectively. The initial model of each nanobody was predicted using SWISS-MODEL (https://swissmodel.expasy.org/), and then fitted into the density map. Several rounds of refinement in Phenix-1.16 [48] and manual building in Coot-0.8.9 were performed until the final reliable models were obtained. Model and map statistics are summarized in S1 Table. Figures were generated using UCSF Chimera X v0.93 [49] and PyMol v2.5.2 [50].

## Supporting information

S1 Fig Flow chart of cryo-EM image processing and 3D reconstruction for the complex of prototypic SARS-CoV-2 spike and Nanosota-5. Representative raw cryo-EM images and 2D classes are presented. 3D refinements using the good particles generated an overall 3.8 Å map with C3 symmetry. The final map, half-map FSC curves, angular distribution plot, and accompanying local resolution illustration are enclosed in the dashed black boxes.
(PDF)

S2 Fig Flow chart of cryo-EM image processing and 3D reconstruction for the complex of prototypic SARS-CoV-2 spike and Nanosota-6. Representative raw cryo-EM images and 2D classes are presented. 3D refinements using the good particles generated an overall 2.8 Å map with C3 symmetry. The final map, half-map FSC curves, angular distribution plot, and accompanying local resolution illustration are enclosed in the dashed black boxes.
(PDF)

S3 Fig Nanosota-7 binds to a non-RBD epitope in S1. The binding interactions between nanobodies (Nanosota-4, -5, and -7) and SARS-CoV-2 spike domains (RBD, S1, and spike ectodomain) were examined using ELISA. PBS buffer was used as a negative control. Nanosota-4 binds to the RBD, whereas Nanosota-5 and -7 both bind to non-RBD regions in S1. Comparisons of target binding between the negative control and nanobodies were performed using an unpaired two-tailed Student’s t-test. Error bars represent SEM (n = 3). ***p<0.001.
(TIF)

S4 Fig Additional data on Nanosota-5’s enhancement of the cell entry of pre-Omicron SARS-CoV-2 pseudoviruses. **(A)** Even at very high concentrations (e.g., 0.4 mg/ml), Nanosota-5-Fc continued to enhance the cell entry of prototypic SARS-CoV-2 pseudoviruses. **(B)** Nanosota-5-Fc enhanced the cell entry of both the alpha and delta variants of SARS-CoV-2 pseudoviruses. Comparisons of pseudovirus entry between conditions with and without Nanosota-5-Fc (i.e., 100% of pseudovirus entry) were performed using an unpaired two-tailed Student’s *t*-test. Error bars represent SEM (n = 3). ***p<0.001; **p<0.01; *p<0.05.
(TIF)

S5 Fig Flow chart of cryo-EM image processing and 3D reconstruction for the complex of SARS-CoV-2 XBB.1.5 spike and Nanosota-5. Representative raw cryo-EM images and 2D classes are presented. 3D refinements using the good particles generated an overall 3.49 Å map with C3 symmetry. The final map, half-map FSC curves, angular distribution plot, and accompanying local resolution illustration are enclosed in the dashed black boxes.
(PDF)

S6 Fig Comparison of Nanosota-5-contacting residues on prototypic and Omicron spikes. The Nanosota-5-contacting residues on prototypic and XBB.1.5 spikes were identified using cryo-EM structures of the respective spike/Nanosota-5 complexes analyzed by PDBePISA (https://www.ebi.ac.uk/pdbe/pisa/). Additionally, the spikes from two other Omicron subvariants, BA.1 and BA.5, were included in the sequence comparisons. # Due to deletions, the residue numbering in the Omicron spikes is three units lower than their corresponding residues in the prototypic spike. For clarity, this difference is not depicted in the figure.
(TIF)

S7 Fig Comparison of surface electrostatic potentials in the Nanosota-5 epitope and adjacent regions. The residues near but outside the Nanosota-5 binding site were analyzed, revealing several differences between the prototypic spike **(A)** and the XBB.1.5 spike **(B)**. This figure was created using PyMol v2.5.2.
(TIF)

S8 Fig Impact of cellular proteases on Nanosota-5’s activities. **(A)** ELISA comparing the binding affinity of Nanosota-5-His for the recombinant prototypic SARS-CoV-2 spike ectodomain, with or without the furin cleavage site. **(B)** Western blot analysis of cells co-expressing Nanosota-5-Fc and either the prototypic or XBB.1.5 spike. The amounts of Nanosota-5-Fc-expressing plasmid used for co-transfection with the spike-expressing plasmid are indicated. The cleavage state of the cell-surface-expressed spike was detected by Western blot using anti-C9 antibodies targeting the C-terminal C9 tag of the spikes. **(C)** Pseudovirus entry into cells co-expressing human ACE2 and TMPRSS2.
(TIF)

S9 Fig Coronavirus entry pathways via viral receptor or Fc receptor (FcR). **(A)** SARS-CoV-2 entry through the viral receptor ACE2. **(B)** Coronavirus entry facilitated by RBD-targeting antibodies and FcR, a molecular mechanism previously identified for antibody-dependent enhancement (ADE) of coronavirus entry (see main text). **(C)** SARS-CoV-2 entry through the viral receptor ACE2, enhanced by non-RBD targeting nanobodies, a molecular mechanism identified in the current study.
(TIF)

S10 Fig Comparison of the entry-enhancing epitope identified in the current study with the entry-enhancing human antibody epitope in the PDB. The PDB ID for the entry-enhancing human antibody epitope is 7DZX (shown in dark gray). The entry-enhancing epitope identified in the current study (shown in blue) partially overlaps with the entry-enhancing human antibody epitope.
(TIF)

S1 Table Cryo-EM data collection, refinement and validation statistics of the prototypic spike/Nanosota-5, prototypic spike/Nanosota-6, and XBB.1.5 spike/Nanosota-5 complexes. (PDF)
