# Supplementary figures and images for: Dual-role epitope on SARS-CoV-2 spike enhances and neutralizes viral entry across different variants
Source: PLoS Pathog. 2024 Sep 5;20(9):e1012493. doi: 10.1371/journal.ppat.1012493 (PMC11407660; doi:10.1371/journal.ppat.1012493)

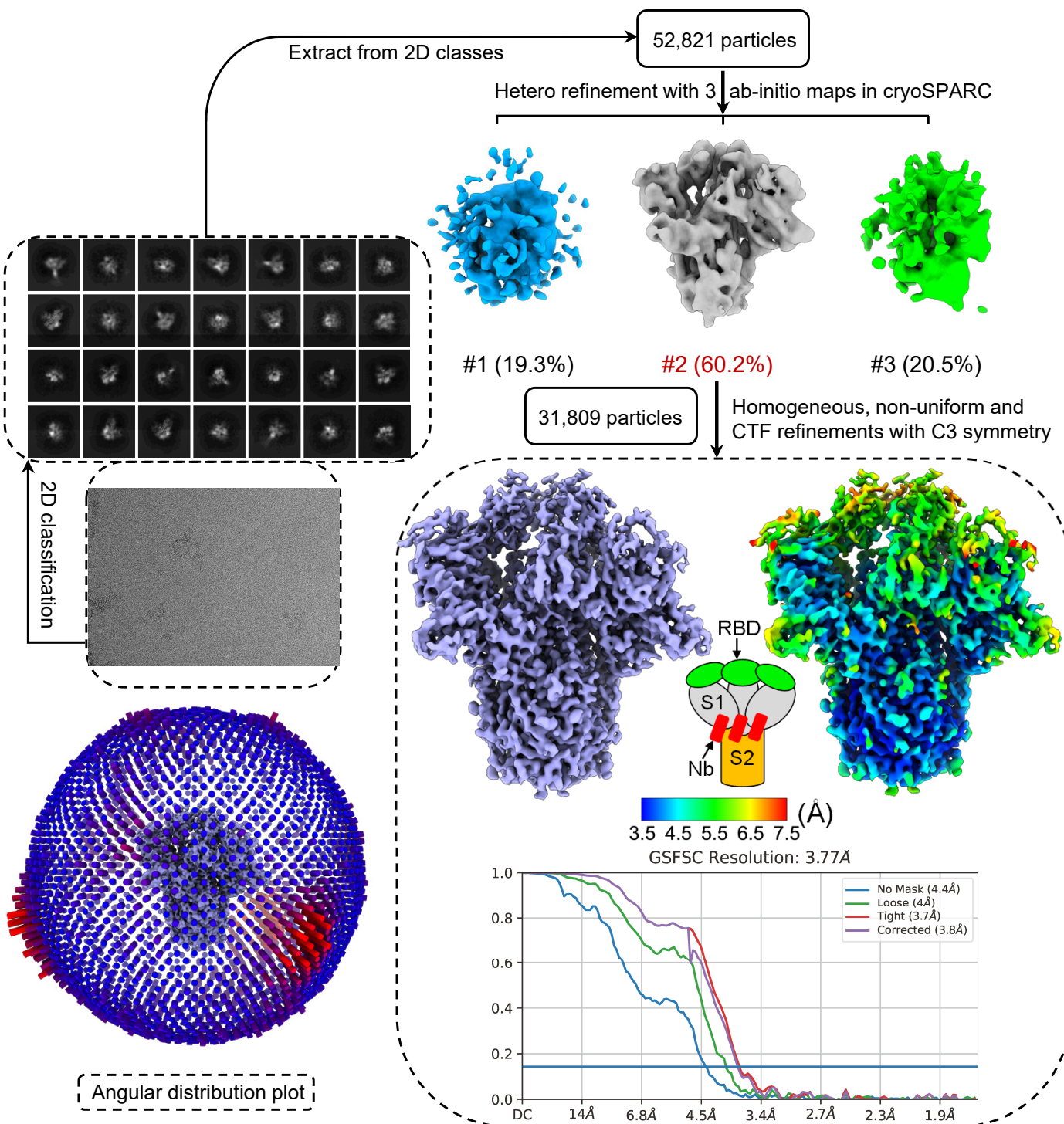

Supplement: S1 Fig — Representative raw cryo-EM images and 2D classes are presented. 3D refinements using the good particles generated an overall 3.8 Å map with C3 symmetry. The final map, half-map FSC curves, angular distribution plot, and accompanying local resolution illustration are enclosed in the dashed black boxes. (PDF) [file ppat.1012493.s001.pdf]

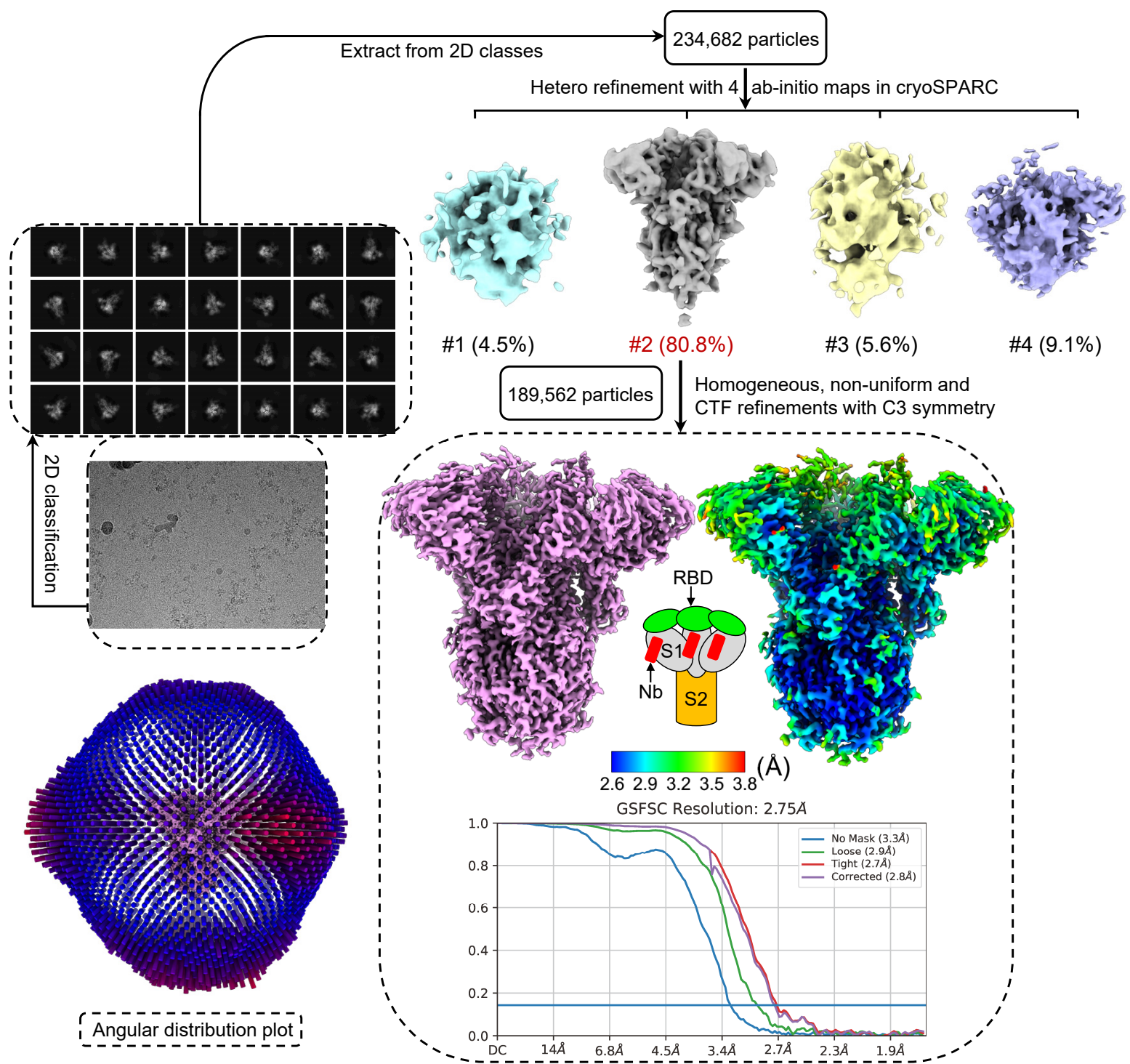

Supplement: S2 Fig — Representative raw cryo-EM images and 2D classes are presented. 3D refinements using the good particles generated an overall 2.8 Å map with C3 symmetry. The final map, half-map FSC curves, angular distribution plot, and accompanying local resolution illustration are enclosed in the dashed black boxes. (PDF) [file ppat.1012493.s002.pdf]

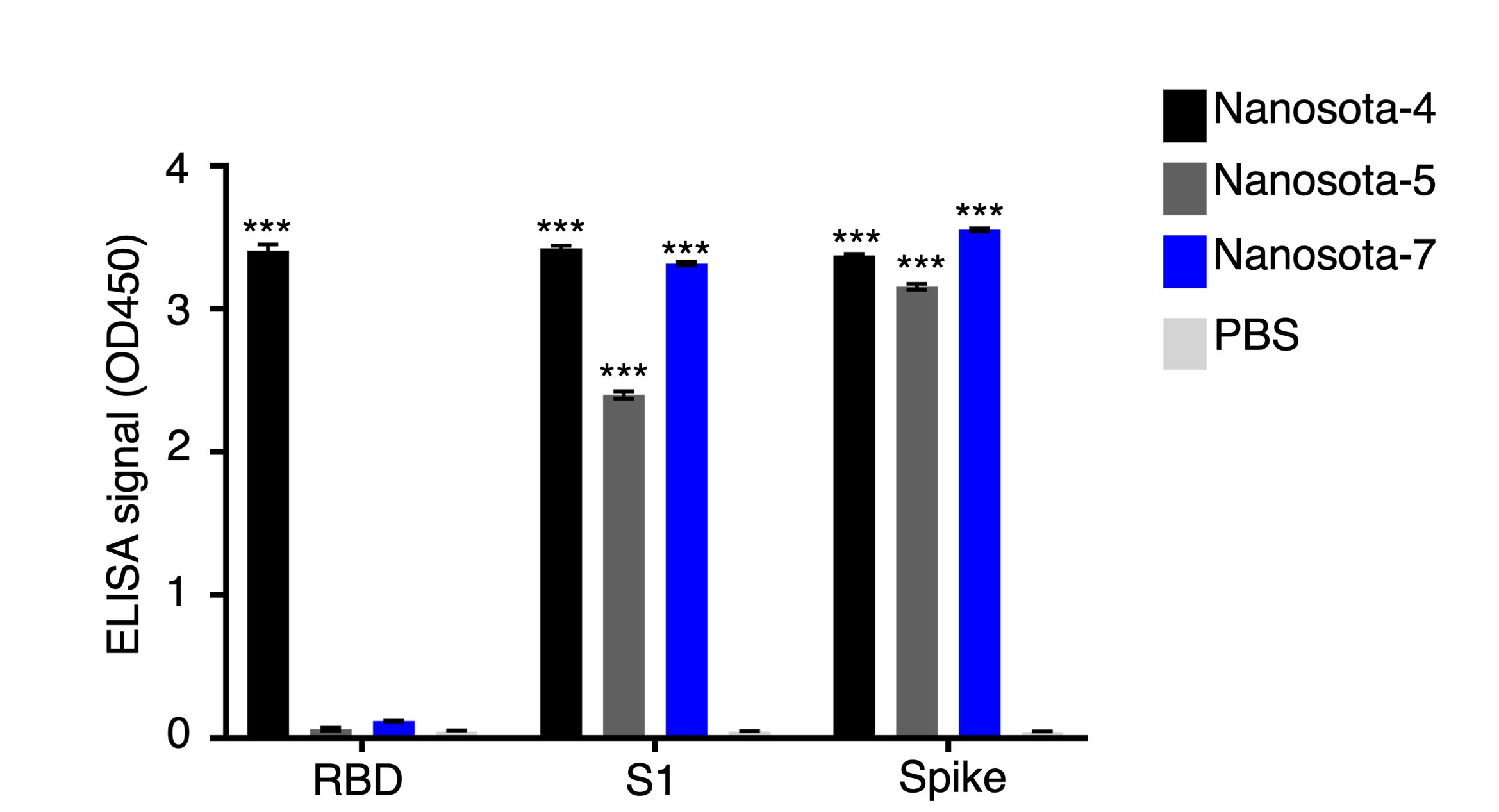

Supplement: S3 Fig — The binding interactions between nanobodies (Nanosota-4, -5, and -7) and SARS-CoV-2 spike domains (RBD, S1, and spike ectodomain) were examined using ELISA. PBS buffer was used as a negative control. Nanosota-4 binds to the RBD, whereas Nanosota-5 and -7 both bind to non-RBD regions in S1. Comparisons of target binding between the negative control and nanobodies were performed using an unpaired two-tailed Student’s t-test. Error bars represent SEM (n = 3). ***p<0.001. (TIF) [file ppat.1012493.s003.tif]

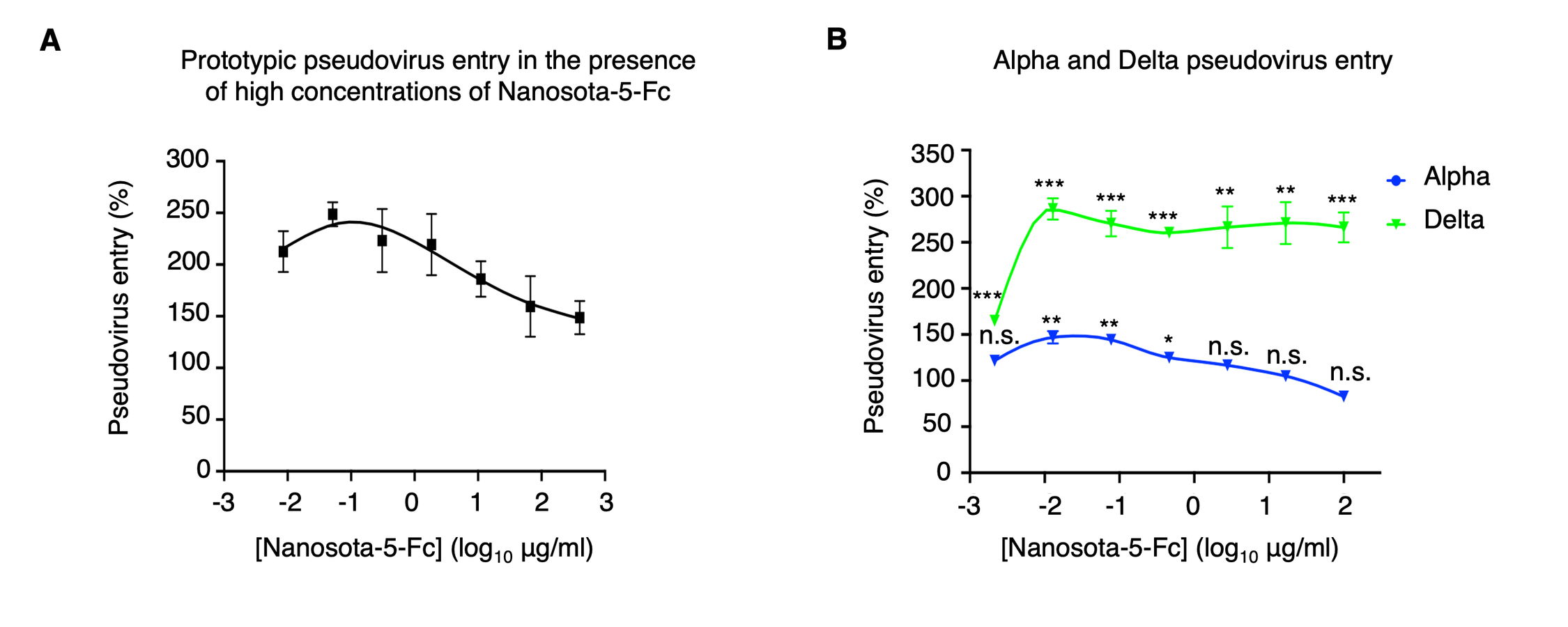

Supplement: S4 Fig — (A) Even at very high concentrations (e.g., 0.4 mg/ml), Nanosota-5-Fc continued to enhance the cell entry of prototypic SARS-CoV-2 pseudoviruses. (B) Nanosota-5-Fc enhanced the cell entry of both the alpha and delta variants of SARS-CoV-2 pseudoviruses. Comparisons of pseudovirus entry between conditions with and without Nanosota-5-Fc (i.e., 100% of pseudovirus entry) were performed using an unpaired two-tailed Student’s t-test. Error bars represent SEM (n = 3). ***p<0.001; **p<0.01; *p<0.05. (TIF) [file ppat.1012493.s004.tif]

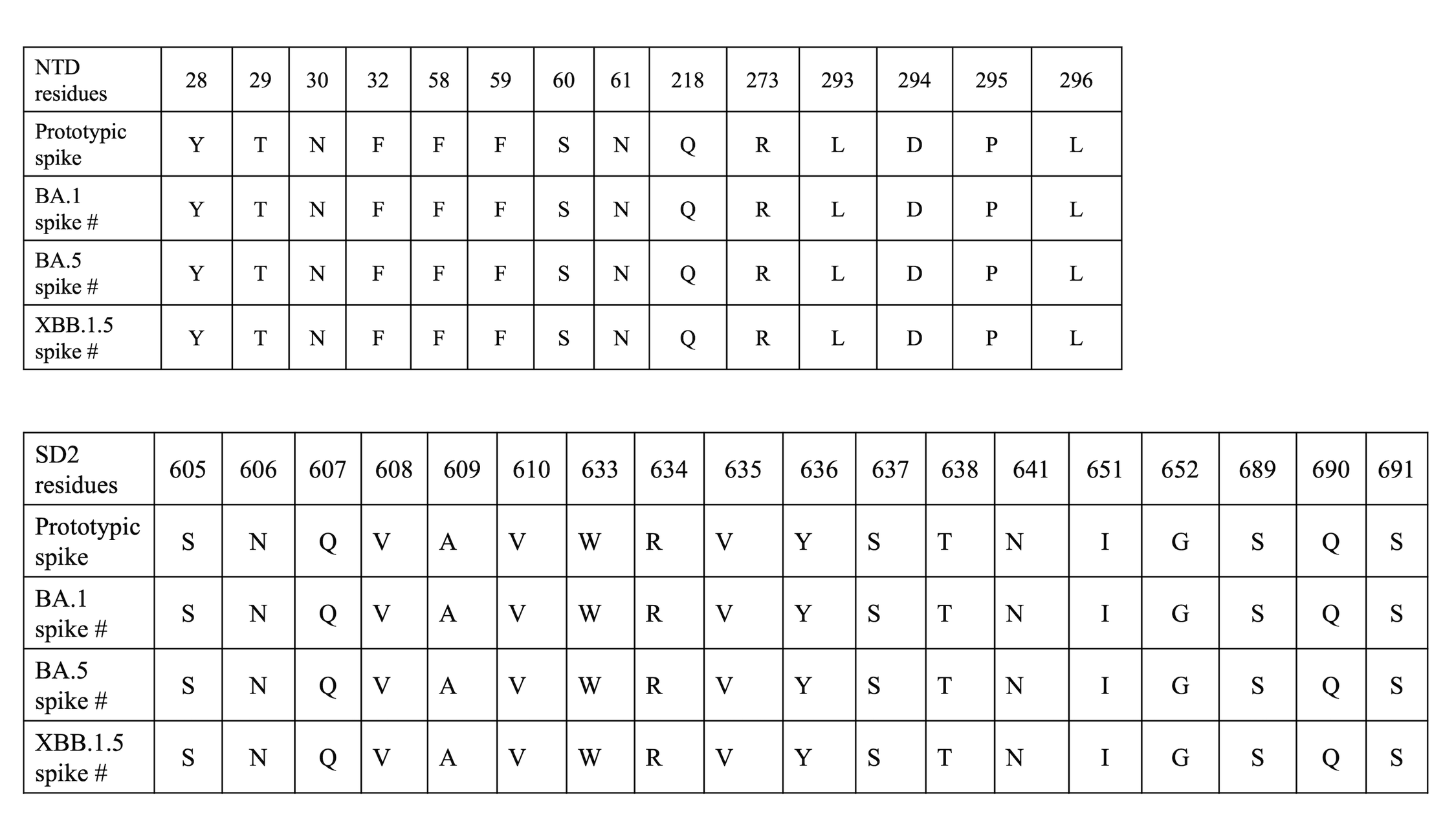

Supplement: S6 Fig — The Nanosota-5-contacting residues on prototypic and XBB.1.5 spikes were identified using cryo-EM structures of the respective spike/Nanosota-5 complexes analyzed by PDBePISA (https://www.ebi.ac.uk/pdbe/pisa/). Additionally, the spikes from two other Omicron subvariants, BA.1 and BA.5, were included in the sequence comparisons. # Due to deletions, the residue numbering in the Omicron spikes is three units lower than their corresponding residues in the prototypic spike. For clarity, this difference is not depicted in the figure. (TIF) [file ppat.1012493.s006.tif]

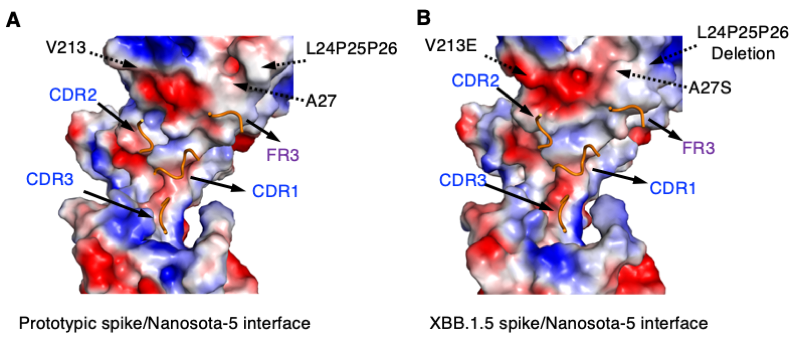

Supplement: S7 Fig — The residues near but outside the Nanosota-5 binding site were analyzed, revealing several differences between the prototypic spike (A) and the XBB.1.5 spike (B). This figure was created using PyMol v2.5.2. (TIF) [file ppat.1012493.s007.tif]

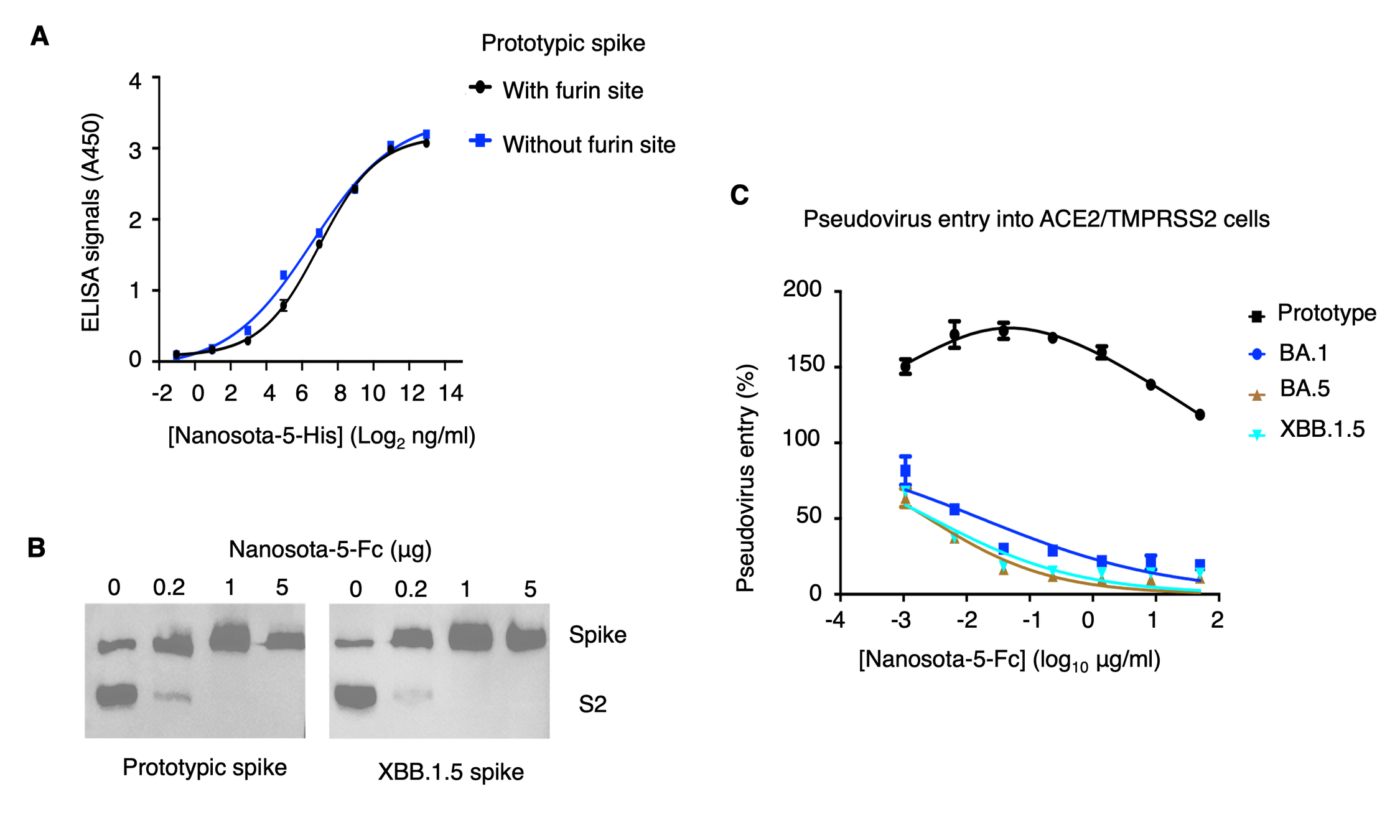

Supplement: S8 Fig — (A) ELISA comparing the binding affinity of Nanosota-5-His for the recombinant prototypic SARS-CoV-2 spike ectodomain, with or without the furin cleavage site. (B) Western blot analysis of cells co-expressing Nanosota-5-Fc and either the prototypic or XBB.1.5 spike. The amounts of Nanosota-5-Fc-expressing plasmid used for co-transfection with the spike-expressing plasmid are indicated. The cleavage state of the cell-surface-expressed spike was detected by Western blot using anti-C9 antibodies targeting the C-terminal C9 tag of the spikes. (C) Pseudovirus entry into cells co-expressing human ACE2 and TMPRSS2. (TIF) [file ppat.1012493.s008.tif]

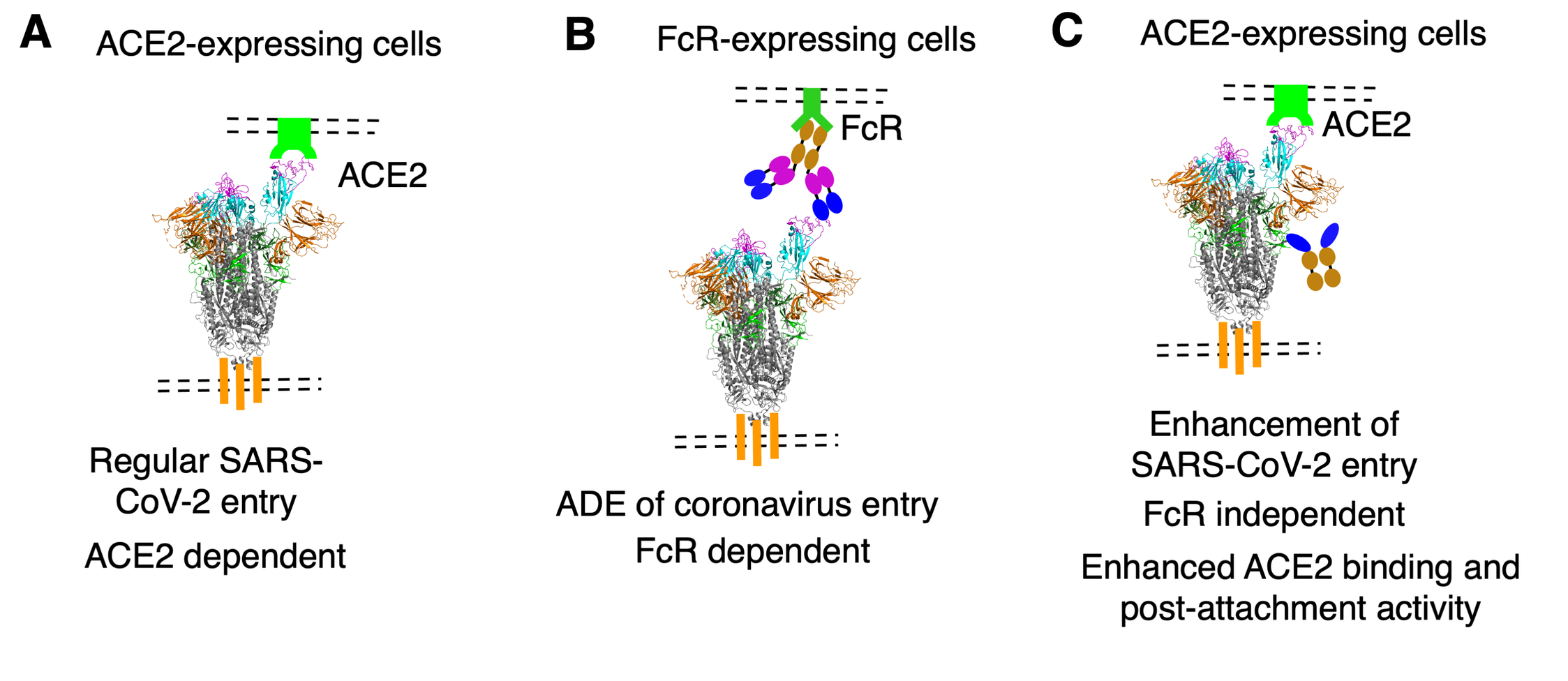

Supplement: S9 Fig — (A) SARS-CoV-2 entry through the viral receptor ACE2. (B) Coronavirus entry facilitated by RBD-targeting antibodies and FcR, a molecular mechanism previously identified for antibody-dependent enhancement (ADE) of coronavirus entry (see main text). (C) SARS-CoV-2 entry through the viral receptor ACE2, enhanced by non-RBD targeting nanobodies, a molecular mechanism identified in the current study. (TIF) [file ppat.1012493.s009.tif]

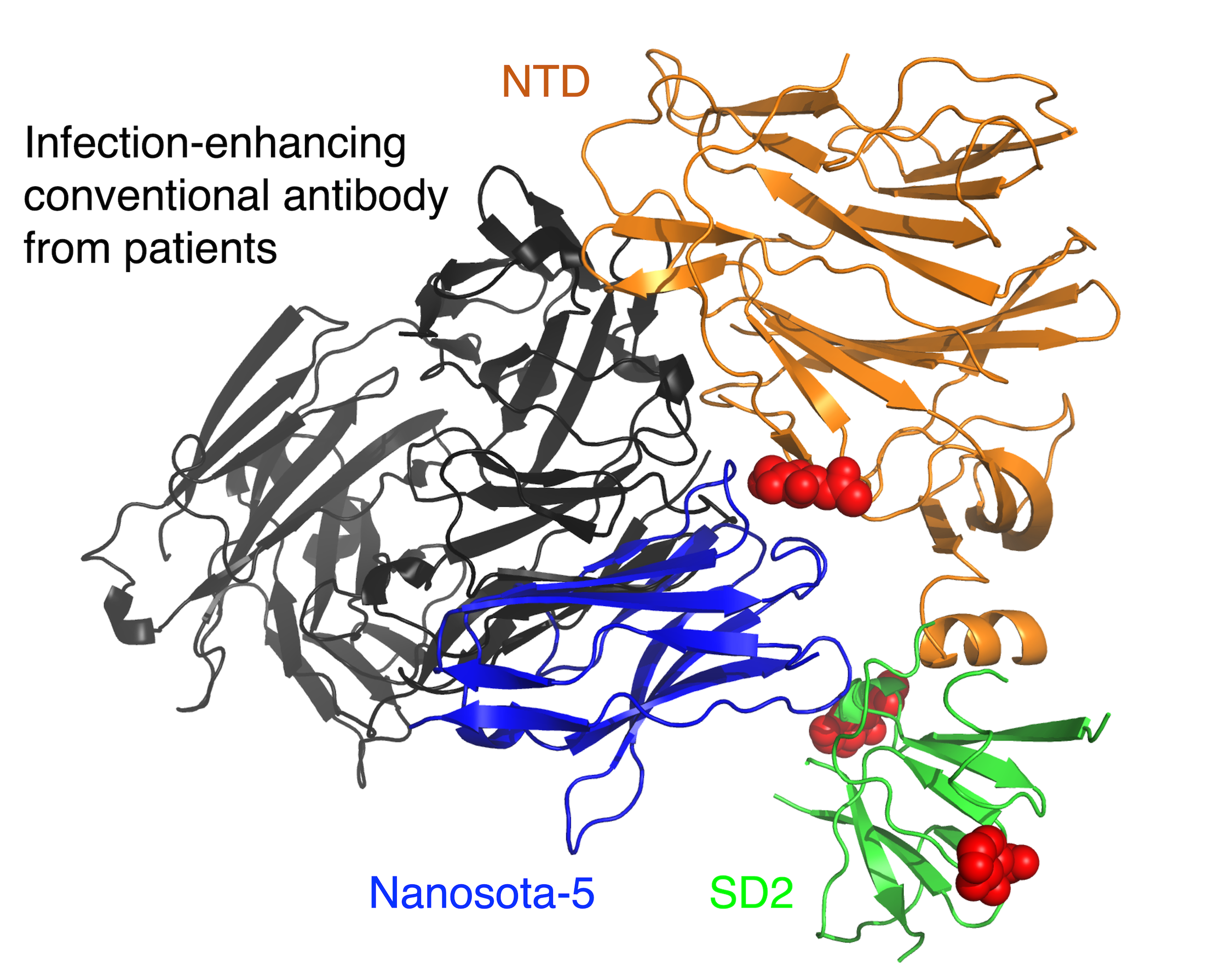

Supplement: S10 Fig — The PDB ID for the entry-enhancing human antibody epitope is 7DZX (shown in dark gray). The entry-enhancing epitope identified in the current study (shown in blue) partially overlaps with the entry-enhancing human antibody epitope. (TIF) [file ppat.1012493.s010.tif]
