# Supplementary material for: Dual-role epitope on SARS-CoV-2 spike enhances and neutralizes viral entry across different variants
Source: PLoS Pathog. 2024 Sep 5;20(9):e1012493. doi: 10.1371/journal.ppat.1012493 (PMC11407660; doi:10.1371/journal.ppat.1012493)
Supplement: S5 Fig — Representative raw cryo-EM images and 2D classes are presented. 3D refinements using the good particles generated an overall 3.49 Å map with C3 symmetry. The final map, half-map FSC curves, angular distribution plot, and accompanying local resolution illustration are enclosed in the dashed black boxes. (PDF) [file ppat.1012493.s005.pdf]

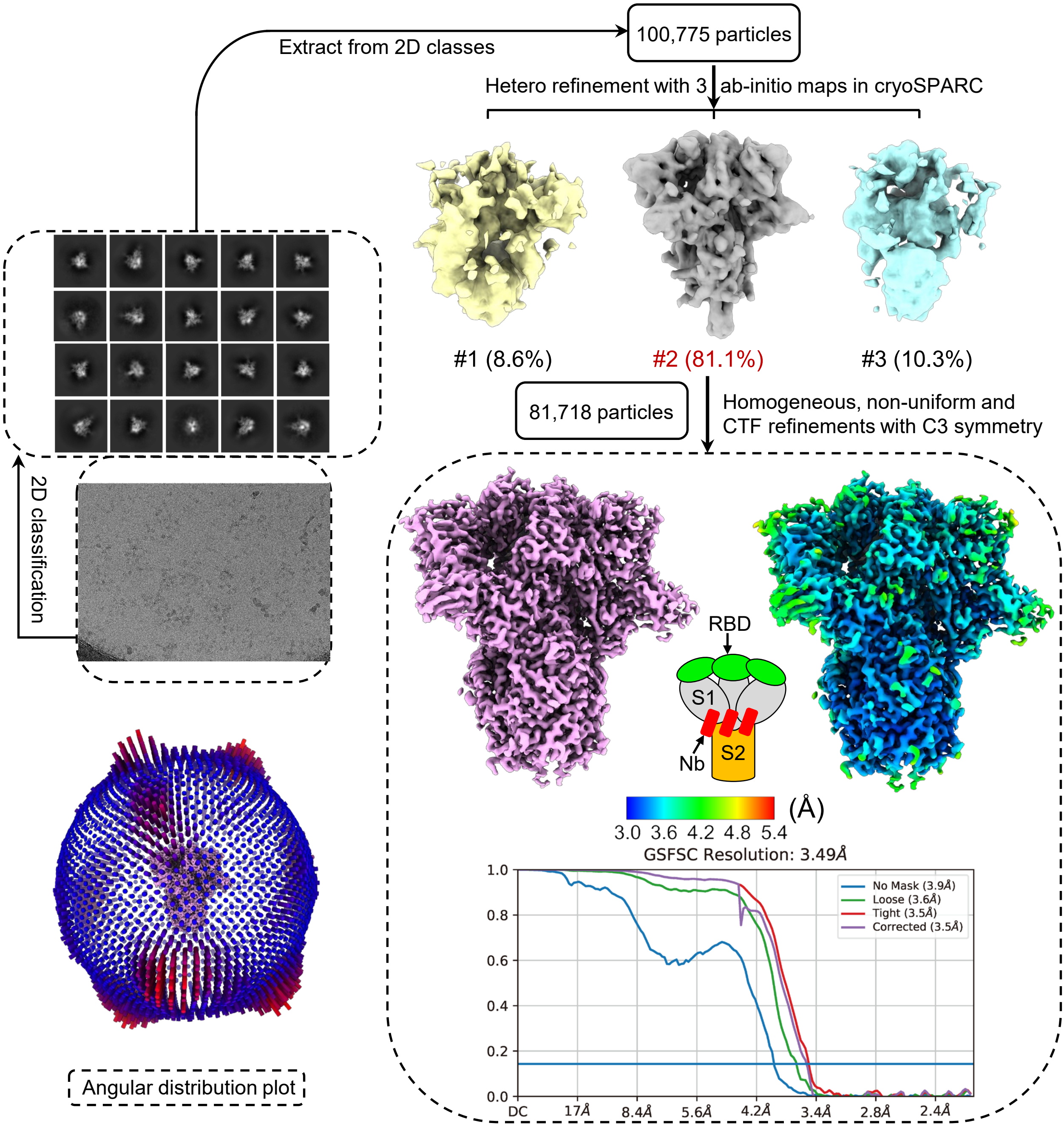

Extract from 2D classes
100,775 particles
Hetero refinement with 3 ab-initio maps in cryoSPARC
#1 (8.6%)
#2 (81.1%)
#3 (10.3%)
81,718 particles
Homogeneous, non-uniform and CTF refinements with C3 symmetry
2D classification
RBD
S1
S2
Nb
(Å)
3.0 3.6 4.2 4.8 5.4
GSFSC Resolution: 3.49Å
No Mask (3.9Å)
Loose (3.6Å)
Tight (3.5Å)
Corrected (3.5Å)
1.0
0.8
0.6
0.4
0.2
0.0
DC
17Å
8.4Å
5.6Å
4.2Å
3.4Å
2.8Å
2.4Å
Angular distribution plot
