# Supplementary material for: Dual-role epitope on SARS-CoV-2 spike enhances and neutralizes viral entry across different variants
Source: PLoS Pathog. 2024 Sep 5;20(9):e1012493. doi: 10.1371/journal.ppat.1012493 (PMC11407660; doi:10.1371/journal.ppat.1012493)
Supplement: S1 Table — (PDF) [file ppat.1012493.s011.pdf]

Table S1: Cryo-EM data collection, refinement and validation statistics of the prototypic spike/Nanosota-5, prototypic spike/Nanosota-6, and XBB.1.5 spike/Nanosota-5 complexes.

	Prototypic spike /Nanosota-5 (EMD-29798) (PDB 8G76)	Prototypic spike /Nanosota-6 (EMD-29799) (PDB 8G77)	XBB.1.5 spike /Nanosota-5 (EMD-42218) (PDB 8UG9)
**Data collection and processing**			
Magnification	130,000	130,000	81,000
Voltage (kV)	300	300	300
Electron exposure (e–/Å^2)	40.00	40.00	50.00
Defocus range (μm)	-0.8 ~ -2.4	-0.8 ~ -2.4	-1.0 ~ -2.0
Pixel size (Å)	0.664	0.664	1.1
Symmetry imposed	C3	C3	C3
Initial particle images (no.)	52,821	234,682	100,775
Final particle images (no.)	31,809	189,562	81,718
Map resolution (Å)	3.8	2.8	3.49
FSC threshold	0.143	0.143	0.143
Map resolution range (Å)	3.5-7.5	2.6–3.8	3.0-5.4
**Refinement**			
Initial model used (PDB code)	7TGY	7TGY	8IOS
Model resolution (Å)	4.0	3.0	3.6
FSC threshold	0.5	0.5	0.5
Model resolution range (Å)	42.5-3.6	54.2-2.7	44.5-3.6
Map sharpening *B* factor (Å^2)	-124.9	-99.8	-106.9
Model composition			
Non-hydrogen atoms	28572	28945	28386
Protein residues	3566	3608	3531
Ligands	53	61	57
B factors (Å^2)			
Protein	234.87	28.98	32.11
Nucleotide			
Ligand	234.03	43.62	62.73
R.m.s. deviations			
Bond lengths (Å)	0.004	0.005	0.003
Bond angles (°)	0.620	0.828	0.553
Validation			
MolProbity score	1.94	1.96	1.57
Clashscore	12.65	11.70	5.59
Poor rotamers (%)	0.42	1.28	0.23
Ramachandran plot			
Favored (%)	95.29	95.77	96.12
Allowed (%)	4.56	4.09	3.82
Disallowed (%)	0.14	0.14	0.06
